# Proteome Stability as a Key Factor of Genome Integrity

**DOI:** 10.3390/ijms18102036

**Published:** 2017-09-22

**Authors:** Sentiljana Gumeni, Zoi Evangelakou, Vassilis G. Gorgoulis, Ioannis P. Trougakos

**Affiliations:** 1 Department of Cell Biology and Biophysics, Faculty of Biology, National & Kapodistrian University of Athens, Panepistimiopolis, 15784 Athens, Greece; sentiljana.gumeni@gmail.com (S.G.); zoievag@biol.uoa.gr (Z.E.); 2 Department of Histology and Embryology, School of Medicine, National & Kapodistrian University of Athens, 11527 Athens, Greece; vgorg@med.uoa.gr

**Keywords:** aggregates, autophagy, DNA damage response, genome instability, Nrf2, oxidative stress, proteostasis, proteasome

## Abstract

DNA damage is constantly produced by both endogenous and exogenous factors; DNA lesions then trigger the so-called DNA damaged response (DDR). This is a highly synchronized pathway that involves recognition, signaling and repair of the damage. Failure to eliminate DNA lesions is associated with genome instability, a driving force in tumorigenesis. Proteins carry out the vast majority of cellular functions and thus proteome quality control (PQC) is critical for the maintenance of cellular functionality. PQC is assured by the proteostasis network (PN), which under conditions of proteome instability address the triage decision of protein *fold*, *hold*, or *degrade*. Key components of the PN are the protein synthesis modules, the molecular chaperones and the two main degradation machineries, namely the autophagy-lysosome and the ubiquitin-proteasome pathways; also, part of the PN are a number of stress-responsive cellular sensors including (among others) heat shock factor 1 (Hsf1) and the nuclear factor erythroid 2-related factor 2 (Nrf2). Nevertheless, the lifestyle- and/or ageing-associated gradual accumulation of stressors results in increasingly damaged and unstable proteome due to accumulation of misfolded proteins and/or protein aggregates. This outcome may then increase genomic instability due to reduced fidelity in processes like DNA replication or repair leading to various age-related diseases including cancer. Herein, we review the role of proteostatic machineries in nuclear genome integrity and stability, as well as on DDR responses.

## 1. Introduction

The genome is continuously exposed to genotoxic attacks, by both endogenous and exogenous factors, which directly or indirectly cause DNA lesions. These lesions can induce several DNA structural changes such as oxidations, depurinations, depyrimidations and single (SSBs) or double (DSBs) strand breaks [1]. Since the nuclear genome harbors almost the entire genetic information of the cell, several mechanisms that preserve genome integrity have been evolved; these mechanisms aim to ensure faithful repair, duplication and inheritance of the genetic material. Cells contain a number of highly coordinated and wired protein machineries that execute DNA replication and/or relaxation-condensation and they also detect and repair the various types of DNA damage. The latter mechanisms are known as the DNA damage response (DDR) pathways and are highly specialized in the recognition of damaged DNA from physiological structural changes. As a result of extensive research it is now understood that the repair pathways activated upon DNA damage largely depend on the type of the lesion. The first step of DDR is the initial identification of DNA damage, followed by the recruitment of DNA repair factors and the effective repair of the lesion [1]. Failure of DDR is often associated with chromosome rearrangements and chromosomal loss that finally lead to genomic instability and predisposition to various types of cancer, developmental defects, infertility, immune deficiency and neurodegenerative disorders [2].

Proteome stability is ensured by a multi-compartmental system that coordinates protein synthesis, folding, trafficking, disaggregation, and degradation; this system is known as the proteostasis network (PN) [3]. Chaperones curate the structure of proteins and assure proper protein folding, while damaged, misfolded and/or aggregated proteins are degraded by the ubiquitin-proteasome (UPP) or the autophagy-lysosome pathways (ALP) [4]. PN activity is also assisted by transcription factors (mostly functioning as stress sensors), chromatin remodelers, structural components, signaling pathways and other auxiliary modules [5]. The efficiency-functionality of the PN affects both genomic and metabolic cellular stability and can be altered transiently or permanently by physiological alterations, exposure to environmental stress and also during ageing. PN components are an integral part of DDR, since they disassemble and remove chromatin associated DDR protein machineries [6]. Perturbation of the PN activity affects the cell buffering capacity and leads to the accumulation of misfolded and/or damaged proteins; thus, its dysfunction is associated with the emergence of chronic diseases caused by protein aggregation, including neurodegeneration, cancer, type II diabetes and heart disease [7]. Herein, we focus on the crosstalk and functional interplay between proteome and genome stability. Furthermore, we discuss how proteotoxic stress and disruption of PN modules functionality impacts on genome stability and integrity.

## 2. DNA Damage Responses

**Figure 1:**
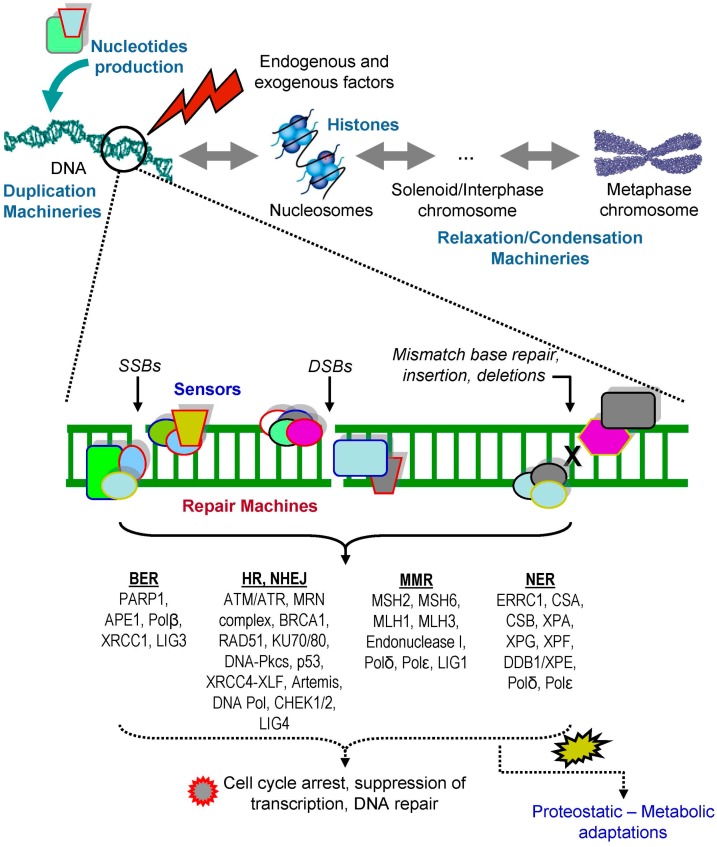
Protein machines in genome maintenance and repair. Optimum functionality of the proteostasis network impacts on all levels of genome stability as it affects both the physiological processes of nucleotides production, DNA duplication and/or relaxation-condensation, as well as DNA damage sensing and repair. Endogenous and exogenous factors can induce DNA damage in multiple ways such as single strand breaks (SSBs), double strand breaks (DSBs), single or short-patch base lesions and DNA base mismatches. SSBs repair is executed via the base excision repair (BER) pathway (involving, among others, PARP1/2 and XRCC1), while DSBs repair mobilizes the homologous recombination (HR; key players here are the ATM/ATR kinases, the Mre11-Rad50-Nbs1 (MRN) complex, BRCA1/2 and RAD51) and the non-homologous end joining (NHEJ; includes Ku70/80, DNA-PKcs and XRCC4-XLF) pathways. Once the cell has sensed DSBs, the DNA repair machinery is recruited to the lesion in relation to the cell cycle stage; in G1 phase cells undergo repair predominantly through the NHEJ repair pathway, whereas in G_2_/M the presence of replicated DNA allows the repair through the HR pathway. The mismatch repair pathway (MMR) is executed (among others) via the MSH2/6, MLH1, PMS2 and Exonuclease 1 proteins, while UV-induced DNA lesions are effectively repaired by the nucleotide excision repair (NER). DDR triggers downstream actions (e.g., via CHEK1, CHEK2 and p53 activation) that suppress transcription and cell cycle progression or trigger apoptosis if the damage is not repairable. Likely DDR also induces a number of proteostatic and/or metabolic adaptations, which remain not well understood. It is thus evident that DNA integrity and stability depends heavily on the functionality of its curating protein machines.

Endogenous sources such as reactive oxygen species (ROS) or metabolic products, along with exogenous factors, such as UV irradiation, chemical agents, lifestyle habits (e.g., smoking) and/or medical treatments can provoke DNA damage; downstream to DNA damage cells activate specific pathways to repair the lesions, known as DDR (Figure 1). Single or short-patch base lesions are repaired by base-excision repair (BER) and DNA base mismatches are corrected by mismatch repair (MMR). On the other hand, UV-induced DNA lesions are effectively repaired by the nucleotide excision repair (NER) pathway, while DNA crosslinks are repaired by the Fanconi anemia (FA) pathway [8]. The whole DDR process of chromatin remodeling and of the chemical reactions involved in lesions removal is tightly controlled by post-translational modifications (PTMs) of the participating polypeptides, including phosphorylation, ubiquitination, sumoylation, methylation and acetylation of protein machineries structural components [9,10,11]. In BER, a base lesion is identified by a DNA glycosylase that catalyzes the cleavage of an *N*-glycosidic bond, removing the damaged base and creating an apurinic or apyrmidinic site, which recruits poly(ADP-ribose) polymerase 1 (PARP1) and DNA apurinic/apyrimidinic (AP) endonuclease 1 (APE1). APE1 removes the deoxyribose phosphate backbone at the site of lesion, generating nicked DNA, enhancing PARP1 ADP-ribosylation activity to keep DNA in an open structure. Other components of BER are also recruited, e.g., protein XRCC1 (X-ray repair cross-complementing protein 1), the DNA end-processing kinase/phosphatase PNK (bifunctional polynucleotide phosphatase/kinase), the gap-filling polymerase DNA polymerase β and DNA ligase III [12]. MMR is a highly conserved pathway that corrects DNA mismatches generated during DNA replication involving heterodimeric complexes [13]. The heterodimer of the MutS homolog (MSH), MSH2–MSH6 (MutSα) or MSH2–MSH3 (MutSβ) binds to mismatched DNA, following the recruitment of a heterodimer of MutL homolog (MLH). Several heterodimers of MutL homologues (MLH) have been identified including MutLα (complex of MLH1 and PMS2), MutLβ (complex of MLH1 and PMS1) and MutLγ (complex of MLH1 and MLH3) [14,15,16]. The MutL protein complex then recruits the necessary proteins for MMR repair [17]. NER recognizes helix-distorting base lesions induced by factors such as UV-induced damage and bulky chemical adducts. There are two distinct NER pathways: the global genome nucleotide excision repair (GG-NER), which detects and eliminates bulky damages in the entire genome, and the transcription-coupled nucleotide excision repair (TC-NER), which operates when damage to a transcribed DNA strand limits transcription activity. GG-NER is controlled by XPC; a specialized protein factor that reveals the damage, while TC-NER is activated by the stopping of RNA polymerase II at the damaged sites of a transcribed strand [18,19,20].

DSBs are considered as the most deleterious DNA lesions, since, if not properly repaired, can lead to chromosomal rearrangement and cytotoxicity [21]. Notably, DSBs can also be generated during physiological cellular processes such as DNA replication, recombination, meiosis or mitosis. DSBs repair involves two types of responses, namely, the homologous recombination (HR) and the non-homologous end joining (NHEJ) pathways. Mammalian cells repair of DSBs is mainly carried out through the NHEJ pathway because of the highly condensed chromatin during G_1_ and G_2_ cell cycle phases. NHEJ is mediated by the combined action of different proteins, such as DNA-PK (composed of the catalytic subunit DNA-PKcs and the regulatory subunit Ku (a heterodimer of Ku70 and Ku80)), XRCC4 (X-ray repair complementing defective repair in Chinese hamster cells-4) and DNA ligase IV (LIG4) [22,23]. Although NHEJ is error-prone, it can operate in all phases of the cell cycle [24]. DNA lesions trigger the activation of various kinases, which constitute the primary transducers of the DDR signaling cascade. Among those, the best studied are the highly conserved ATM (ataxia telangiectasia mutated) and ATR (ATM and Rad3 related). The phosphatidylinositol 3-kinase-related kinases (PIKKs) and the DNA-dependent protein kinase catalytic subunit (DNA-PKcs). Upon DNA damage the Mre11-Rad50-Nbs1 (MRN) heterotrimeric complex binds to DSBs and induces the auto-phosphorylation of ATM [25,26], while cofactor ATRIP (ATR interacting protein) and the binding of the Rad9-Hus1-Rad1 (9-1-1) clamp complex (upon replication stress, collapsed DNA replication forks and UV damage) activate ATR [27,28].

ATM or ATR activation leads to the phosphorylation of many (more than 700) downstream substrates involved in DNA repair, inhibition of cell cycle progression and/or apoptosis. Many of the ATM activated proteins are also targets of ATR and DNA-PK, suggesting a redundancy in this pathway [29,30]. The formation of γ-H_2_AX foci is the initial signaling event in the activation of DNA damage checkpoint pathways, and probably the best biomarker of DNA damage. H_2_AX is phosphorylated at Ser-139 by ATM/ATR or DNA-PK in the chromatin flanking the damage site. The ATM/ATR-dependent phosphorylation of H_2_AX is important for the recruitment and accumulation in the DNA damage site of MDC1 (mediator of DNA damage checkpoint 1), which is the master regulator of the microenvironment close to the lesion and enables the formation of breast cancer type 1 susceptibility protein (BRCA1) and p53-binding protein 1 (53BP1) foci [31,32]. In order to amplify and further transmit the checkpoint signals, ATM phosphorylates checkpoint kinase-2 (CHEK2) at Thr-68, whereas ATR phosphorylates checkpoint kinase-1 (CHEK1) at two residues, i.e., Ser-317 and Ser-345 [33,34]. The activation of checkpoint kinases triggers a phosphorylation cascade that affects (among many other factors) the tumor suppressor p53, which turns on the transcription of downstream targets, leading to arrest of cell cycle, DNA repair and/or senescence [33,35]. The role of p53 in cell survival and cell death is mediated by the positive and negative feedback loops of the ATM/ATR phosphorylation of p53 Ser-15, as well as on phosphorylation of p53 Ser-20 by CHEK1/CHEK2. These PTMs stabilize p53 by preventing its binding to MDM2 (mouse double minute 2 homolog). p53 is also uncoupled from MDM2 after phosphorylation of Thr-18; then it translocates to the nucleus where it mediates the activation of its transcriptional targets [36]. Interestingly, MDM2 seems also to be inhibited by a direct ATM and ATR phosphorylation on Ser-395 and Ser-407, suggesting a multiple control of these kinases on p53 function [37,38]. Worth mentioning is that p53 has low affinity for the genes involved in apoptosis, and high affinity for genes that mediate cell cycle arrest. Thus, it is suggested that p53 activity is mainly defined by the entity of DNA lesions, i.e., high levels of damage continuously induce the p53 activity, while low damage levels only transiently activate p53 [39]. If the DSB is repaired, either by NHEJ or HR, the checkpoint response is inactivated and this output then signals the downregulation of the DNA damage checkpoint genes [40]. Unrepaired DNA damage, dysfunction of DDR and/or altered expression of DDR genes, lead to extensive genome instability, which is a well-known hallmark of cancer [41,42].

## 3. The Proteostasis Network

Considering that most of the critical cellular functions are performed by sophisticated protein machines [43] it is not surprising that proteostasis (proteome homeodynamics) is critical (despite the fact that species with hugely different life spans express highly homologous proteins with very similar half-lives) for cellular functionality and consequently for the overall healthspan and survival of the organisms. Proteome integrity is maintained by the PN, a multi-compartmental highly wired system that coordinates protein synthesis, folding, trafficking, disaggregation and degradation [3,44]. Key components of the PN are the protein synthesis and trafficking modules, the endoplasmic reticulum unfolded protein response (UPR^ER^), the molecular chaperones and the two main degradation machineries, namely the ubiquitin-proteasome (UPP) and the autophagy-lysosome (ALP) pathways. UPR^ER^ is a cellular stress response system, which is highly conserved among species and is triggered by increased unfolded or misfolded polypeptides in the ER lumen. The main goal of UPR^ER^ is to restore proteome stability by either attenuating de novo protein synthesis or by inducing expression of chaperones in order to restore proper folding; if protein functionality cannot be repaired, UPR^ER^ leads cells to apoptosis [45]. In mammalian cells, the three major pathways that are implicated in UPR^ER^ are the protein kinase R (PKR)-like endoplasmic reticulum kinase (PERK)/activating transcription factor (ATF4); the inositol-requiring enzyme 1 (IRE1)/X box-binding protein 1 (XBP1) and the activating transcription factor 6 (ATF6) [46]. Molecular chaperones, which are also known as heat shock proteins (Hsps), are a diverse family of proteins that are responsible for proper protein folding, unfolding and remodeling; consequently, chaperones curate proteome in order to maintain polypeptides function and structure [47]. Chaperones are central for the PN function and can be broadly grouped into, the Hsp70, Hsp90, DNAJ/Hsp40, chaperonin/Hsp60 and small Hsp (sHsp) families [48].

UPP is composed of the ubiquitin-conjugating enzymes and the 26S proteasome, and it degrades short-lived polyubiquitinated normal proteins (e.g., nucleo-cytosolic regulatory proteins) and non-functional or misfolded polypeptides [3]. Oxidized proteins are likely degraded by the proteasome in an ubiquitin-independent manner [49,50]. The 26S proteasome consists of a catalytic 20S core particle bound to 19S regulatory particles [4,51]. Consequently, UPP ensures a number of cellular processes, such as development, immune responses, metabolism, signal transduction, cell cycle progression, as well as cell death [3]. Moreover, UPP is actively involved in the degradation of mitochondrial fusion/fission proteins [52,53,54] and thus, apart from curating genome (see below) and proteome stability, it is also actively involved in regulation of mitostasis. On the other hand, ALP is mostly involved in the degradation of long-lived proteins, aggregated ubiquitinated proteins, polypeptides modified by non-enzymatic post-translational modifications, macromolecules and cytosolic portions; it also functionally involved in the recycling of damaged organelles via lysosome [55,56,57]. In mammalian cells, the most studied forms of autophagy are macroautophagy, microautophagy and chaperone-mediated autophagy [58,59,60]. ALP is subject to regulation by several metabolic signaling pathways, including adenosine monophosphate-activated protein kinase (AMPK), Sirtuin 1 (SIRT1) and TOR [55].

Parts of the PN are also several short-lived transcription factors that collectively function as stress sensors and mobilize genomic cytoprotective responses upon increased amounts of stressors. These (among others) include p53 that mostly mobilizes cellular responses upon genomic instability [61], heat shock factor 1 (Hsf1) that regulates the levels of molecular chaperones [62], forkhead box O (FoxO) that promotes antioxidant and metabolic genomic responses [63] and also nuclear factor erythroid 2-related factor 2 (Nrf2) that responds to oxidative, electrophilic and/or proteotoxic stress [3,64,65].

The expression level of the PN modules is highly variable, as in order to ensure efficient protection against acute and/or chronic proteotoxic stress, may increase globally or in a specific cellular compartment indicating thus that PN regulation is a highly dynamic process [44]. The PN plasticity is provided by the aforementioned dedicated stress-responsive transcription factors with distinct and complementary transcriptional targets. Therefore, proteome instability activates PN in order to rescue, when attainable, or degrade unfolded, misfolded, damaged and/or non-native polypeptides, a process known as the triage decision of *hold*, *fold*, or *degrade* [66]. Nevertheless, accumulation of protein aggregates, deregulation of protein synthesis and folding or loss of protein clearance mechanisms trigger proteome instability, which is considered a major risk factor for a broad range of (mostly age-associated) diseases including neurodegeneration, cancer, as well as immunological and metabolic disorders [7,43,67]. 

## 4. Endoplasmic Reticulum Unfolded Protein Response (UPR^ER^) in Genome Instability

Accumulation of unfolded and/or misfolded proteins disrupts ER homeostasis leading to ER stress and activation of various intracellular signaling pathways, collectively known as unfolded protein response (UPR) [68,69,70]. Activation of UPR^ER^ triggers (see also above) three main responses: (1) the inhibition of protein synthesis, (2) the induction of genes such as the ER chaperones to increase the protein-folding capacity of the organelle, and (3) the up-regulation of the so called ER-associated protein degradation (ERAD) [71,72]. UPR^ER^ is mainly a cytoprotective response. However its excessive or prolonged activation may result in cell death [72]. The UPR^ER^ pathways are activated in a variety of tumor types and are essential for solid tumors cell survival in an unfavorable environment [70,73,74]. In addition, evidence suggests that the UPR^ER^ is an important mechanism required for cancer cells to maintain malignancy [70,74], while other studies have shown that ER stress induces chromatin changes [72,75,76,77,78,79,80,81,82,83,84,85,86,87,88,89,90]. 

Several studies have linked ER stress to DDR. Yamamori et al. [72] showed that ER stress induced by tunicamycin treatment or glucose deprivation, results in decreased DSB repair and enhances radiosensitivity of tumor cells through the downregulation of RAD51. The proteasomal degradation of RAD51 triggered by ER stress is considered responsible of DSB repair suppression after ionizing radiation (IR) [72]. On the other hand, downregulation of PERK enhances DNA damage repair in irradiated cancer cells [75]. Interestingly, it has been reported that PERK regulates the stability of cyclin D_1_ [76], which mediates the progression of cell cycle from G_1_ to S phase [77] and its overexpression has been reported to promote resistance to IR by upregulating DDR [78]. Moreover, the transcriptional activation of GRP78 has been used extensively as an indicator of UPR^ER^ activation. The induction of GRP78 confers protection against ER stress due to its anti-apoptotic properties and represents the survival arm of UPR^ER^ [79,80,81,82]. Baumeister et al. [83] found that ER stress increases H4 acetylation and GRP78 expression by recruiting the histone acetyl-transferase p300 to the GRP78 promoter. The recruitment of arginine histone methyltransferase, PRMT1, also increases the expression of GRP78 [83]. It is also suggested that PRMT1 mediates the arginine methylation of MRE11 and thus it modulates the activity of the MRN complex [84]. Therefore, ER stress can promote DDR and repair though increased transcription of GRP78. Hypoxia and heat shock also result in chromatin remodeling and ER stress that consequently impact on DDR [85]. ER stress enables the expression of HIF-1 mediated response after the deacetylation and methylation of histones in the proximity of the involved genes [86,87,88] triggering thus the hypoxia adaptive response [89,90]. Reportedly, hypoxia can induce cellular transformation through defective DNA repair and consequently genomic instability [90]. Although further studies are needed in order to clarify the crosstalk of ER stress machineries with DDR, it is evident that UPR^ER^ is implicated in chromatin remodeling affecting thus DNA stability and/or conformation.

## 5. Oxidative Stress in Genome Integrity

ROS are chemically reactive molecules that have essential functions in living organisms like modifying the structure of proteins, activating transcription factors and modulating genes expression. Moderate levels of ROS can promote cell proliferation and differentiation, whereas excessive amounts induce a cellular condition known as oxidative stress [91,92]. In addition, increased oxidative load can damage most (if not all) biomolecules including proteins, DNA (nuclear and mitochondrial) and lipids. ROS, normally, function as intracellular messengers that orchestrate unique signaling events mediated by kinases, ubiquitinases and acetyltransferases. Cellular redox state is maintained via the equilibrium of ROS production and scavenging, which protects macromolecules from radical damage [93,94]. 

Cells adapt to increased amounts of oxidants by activating redox-sensitive transcription factors, such as Nrf2, nuclear factor-κB (NF-κB), c-Jun and hypoxia-inducible factor 1 (HIF-1), which increase the expression of antioxidant molecules (e.g., superoxide dismutase (SOD), catalase and thioredoxin) [95]. Nrf2 activity is regulated by a negative feedback mechanism of Keap1 (Kelch ECH associating protein 1) [96]. Under physiological conditions, Nrf2 is a short-lived protein, because it is constantly targeted by Keap1 for Ub-dependent degradation; parallel to Keap1, the b-TRCP/Gsk-3 axis can also mediate degradation (and thus inhibition) of Nrf2 [97]. In response to increased amounts of oxidants, the three cysteine residues Cys-151, Cys-273 and Cys-288 of Keap1, induce conformational changes in the molecule leading to reduced Nrf2 ubiquitination. This event stabilizes Nrf2, which then translocates to the nucleus to form a heterodimer with its partner Maf (v-Maf avian musculoaponeurotic fibrosarcoma oncogene homolog) and regulate the transcription of its target genes [98,99,100]. Nrf2 binds to the antioxidant response elements (AREs) or electrophile response elements (EpREs) on the DNA and regulates the expression of numerous genes [98]. Notably, Nrf2 also promotes the expression of chaperones and proteasome subunits [65,101,102] and thus it has multiple roles in also maintaining proteome stability.

Oxidative stress represents one of the major factors of DNA damage and downstream instability. It is hypothesized that approximately 10,000 DNA alterations are generated per mammalian cell per day due to oxidants [103,104]. More specifically, oxidative DNA damage includes base (purine and pyrimidine) oxidation, formation of apurinic or apyrmidinic sites, SSBs, DSBs, DNA intra-strand crosslinks, protein-DNA crosslinks and mismatched pairs with damaged bases [105]. Guanine is the most affected base by ROS because of its low redox potential, and the main products of its oxidation are 8-hydroxyguanine and 8-hydroxydeoxyguanosine (8-OHG and 8-OHdG) [106]. Both these products can match with cytosine and adenine, thus leading to GC-to-AT, and therefore are considered highly mutagenic and carcinogenic; 8-OHG and 8-OHdG are commonly used as markers of DNA oxidative lesions [106]. Increased ROS hyperactivate PARP1, which participates in SSB repair and BER. In addition, activation of PARP1 upon ROS-induced DNA damage leads to NAD^+^ consumption and ATP depletion, and therefore activates autophagy via AMPK pathway in order to provide energy for the DDR [107,108]. 

Several evidences show that cancer cells are characterized by elevated ROS levels, and that enhanced oxidative stress is likely a hallmark of tumorigenesis [109,110]. It is not thus surprising that (as shown by several studies) the Nrf2 signaling pathway is constitutively activated in various types of cancer; one of the factors that drive Nrf2 activation in tumors is the accumulation of the p62/SQSTM1 (Sequestosome-1) protein that disrupts the association of the Keap1–Nrf2 complex [111]. p62/SQSTM1 contains a STGE-binding motif (similar to the Nrf2 ETGE motif) and therefore by binding to Keap1, disrupts the Keap1–Nrf2 complex. In addition, a mouse liver-specific autophagy-deficient model that develops adenoma shows increased levels of Nrf2; of Nrf2 transcriptional targets, and of protein aggregates [112], indicating increased proteome instability. The cellular responses to stress involve regulatory changes in many processes, including transcription, mRNA processing and translation. Recently, it has been reported that loss of Nrf2 affects the translational machinery and stimulates mRNA translation in pancreatic cells in a ROS-dependent way [113]. In addition to Nrf2, p53 has also been shown to suppress ROS levels by regulating the expression of several antioxidant genes, including *sod2*, *gpx1* (glutathione peroxidases 1) and *catalase*, or through the indirect activation of TP53-inducible glycolysis and apoptosis regulator (TIGAR) [114,115]. Nrf2 protects colonic epithelial cells from IR, in part by enhancing signaling of the DNA damage response [116]. Loss of Nrf2 in mouse embryonic fibroblasts (MEFs) promotes their immortalization (due to an early loss of *p53* and p53-dependent genes expression) and enhances genomic instability [117]. Studies have demonstrated that p53 regulates negatively the transcription of Nrf2 target genes (e.g., *NAD(P)H guinone dehydrogenase 1* (*ngo1*), and *glutathione S-transferase 1* (*gst1*)) [118]. On the other hand, p53 mutant forms (pan-p53^mut^) enhance transcription of numerous proteasome subunit genes [119]. Notably, Nrf2 is positively regulated by p21 (a downstream target of p53) as it associates with the DLG motif of Nrf2, disrupting the binding of Keap1 [120]. Thus, both the p53 and Nrf2 regulatory axes likely converge on p21 to also modulate antioxidant responses. Interestingly, we recently reported that chronic p53-independent p21 expression causes genomic instability by deregulating replication licensing [121].

Taken together these studies indicate that Nrf2 regulates antioxidant responses, proteostasis and genome integrity; nevertheless, it is still unclear whether the effects on genome integrity are a starting event or a consequence of Nrf2 activity.

## 6. Impact of Molecular Chaperones Function on Genome Stability

Molecular chaperones enable the folding and/or the assembly of other macromolecular structures (e.g., protein machines). One of the principal functions of chaperones is to preserve the folding of the newly synthesized polypeptides preventing thus their aggregation [122]. Small heat-shock proteins (sHsp) [123,124], Hsp60, Hsp70 [125], Hsp110 [126], nucleosome assembly protein histone chaperones [127] and peroxiredoxins [128] have all been associated to genome stability, suggesting that chaperone function is not solely limited to protein folding [129]. In mice, the Hsp genes *hsp70.1* and *hsp70.3* are induced by both endogenous and exogenous stressors, such as heat and toxic substances. The *hsp70.1/3^−/−^* mice displayed genomic instability that is enhanced by heat treatment; also, cells from *hsp70.1/3^+/−^* mice showed a higher frequency of chromosome end-to-end associations. Exposure of those cells to IR leads to more residual chromosome aberrations, radio resistant DNA synthesis (a hallmark of genomic instability), increased cell death and enhanced IR-induced oncogenic transformation [125]. Heat treatment prior to IR exposure enhances cell death and S-phase-specific chromosome damage in *hsp70.1/3^−/−^* cells compared to *hsp70.1/3^+/+^* cells. Also, both in vivo and in vitro studies demonstrate that *hsp70.1* and *hsp70.3* expression alterations result in genome instability under stress conditions [125]. 

p97/VCP (also named, Cdc48) is an evolutionarily conserved AAA-ATPase that is abundantly expressed in cells and is involved in several functions in cells, including, protein degradation, and disassembly, as well as chromatin remodeling. The role of p97/VC in extracting and degrading proteins from chromatin following DNA repair is considered highly important in preserving genome stability. Reduced p97/VCP activity leads to the accumulation of ubiquitinated substrates on chromatin and activation of protein-induced chromatin stress (PICHROS), which leads to genome instability and genotoxic stress. PICHROS affects several cellular processes such as DNA replication, transcription rates and also DDR [130,131,132,133,134,135]. Moreover, studies in yeast and *C. elegans* have showed that p97/VCP is an essential factor for cell cycle progression, since *cdc48* mutations cause arrest or delay transition of cell cycle phases and/or activation of replication checkpoints [136,137].

Another key component of the molecular chaperones family is Hsp90. Hsp90 inhibition prevents BRCA2 (breast cancer type 2 susceptibility protein) from being accumulated at sites of damage, which in turn signals RAD51 to participate effectively in homologous recombination [138,139]; moreover, Hsp90 inhibition causes contraction of human CAG repeats in vivo [138]. Hsp90 also interacts directly with DDR molecules, such as CHEK1. This kinase (see also above) is recruited to sites of DNA damage and delays cell cycle progression, prevents origin firing, stabilizes stalled replication forks, and activates FA complex for DNA repair [140]. Another role of the Hsp90 chaperone machinery is to maintain chromosome transmission fidelity through its indirect participation in kinetochore assembly. Also, Hsp90-mediated assembly of kinetochores implies a potential link between cell stress and chromosome instability [141].

Overall, it is obvious that molecular chaperones functionality is paramount for not only the maintenance of proteome stability, but also for genome integrity. Nevertheless, more work has to be done in order to illuminate the molecular mechanisms and the functional involvement of chaperone protein machines on age- and/or disease-associated-genome instability.

## 7. Ubiquitin-Proteasome Pathway (UPP) and Genome Integrity

The cellular genome is a very complex landscape, and based on the cell type and cell function, genes expression profile can be remarkably different [142]. Supervised protein degradation allows rapid, and irreversible, turn-off of a protein’s function; this process is of critical importance for cellular function since there is a plethora of genome curation and stability related proteins (e.g., transcription factors and protein machines of replication complexes and cell division cycle) that need to be eliminated by the cell through specific processes at the right moment in order to maintain genome integrity and eventually cellular homeodynamics and survival.

These polypeptides are degraded by the 26 proteasome, through the ubiquitin-dependent signaling. Ubiquitin (Ub) is a small highly conserved among the eukaryotes 76 amino acid protein, which is attached to proteins as either a monomer or as a polyubiquitin chain by an enzymatic reaction [143,144]. The conjugation of Ub to the polypeptide is mediated by a series of ligases known as Ub-activating enzymes, namely E1, E2 and E3 ligases. The E1 and E2 enzymes activate the ubiquitin in an ATP-dependent process, while the E3 ligase performs the final step ligating the carboxyl group of the C-terminal of Ub to the target protein. Degradation of the targeted protein by the proteasome requires (mainly) polyubiquitination at lysine 48 [143,144]. It is worth mentioning that due to high numbers (~one million/cell) of existing cellular proteasomes that respond to degradation demands [145], and despite the high rates of protein destruction, it is estimated that the proteasomes are under-loaded during normal conditions, and that proteins accumulate in the cells only after more than an half (~60%) of total cellular proteasomal activity is shut down [146,147]. As mentioned proteostasis is achieved by the interaction and crosstalk of several processes like control of transcription, processing and degradation of mRNAs, translation, protein localization, post-transcriptional and also programmed degradation [148]; thus UPP-mediated degradation is crucial for cell survival and adaptation. Specifically, cells respond to extracellular signals, as well to their metabolic and energetic demands, by adapting their transcription levels, translation capacity and eventually their proteome content. These changes in protein levels are not an immediate process, but there is a timing delay between the transcriptional induction and the increase of protein levels [149]. Nevertheless, high translation rates are made in a fidelity cost, which lead to amino acids incorporation errors that cause conformational changes and, therefore, the formation of non-functional polypeptides that need to be rapidly eliminated via high protein turnover [150].

Genome stability in mitotic cell lineages requires precise (once per cell cycle) DNA replication. In order to avoid re-replication, cells need to suppress licensing of newly replicated DNA until late mitosis. Proteasomal degradation of DNA replication machineries is one of the mechanisms that prevent re-licensing [151,152]. Another important function of UPP is the maintenance of nuclear homeostasis as several studies have shown that proteasome is responsible for degradation of ribosomal proteins and oxidatively damaged histones [153,154]. Although it has been described that aggregates in the nucleoplasm are degraded by the proteasome, it remains unclear whether this is an in situ reaction or protein aggregates are transported for degradation to the cytosol [155]. Furthermore, besides preserving the cellular microenvironment, UPP activity also defines the cell fate by preserving the balance between pro-apoptotic and anti-apoptotic signaling pathways [156]. Cell cycle progression is also an UPP regulated event as the levels of all key cell cycle progression players, e.g., cyclins or cyclin-dependent kinases (CDKs) are regulated by UPP dependent degradation [157].

Considering all the above UPP functions along with the deterioration of its functionality during ageing [3], the association of UPP with pathological disorders is to be expected. Specifically, it has been hypothesized that tumor cells have chronically elevated levels of proteotoxic stress [158]. That is because cancer cells acquire their proliferative capacity in the absence of growth signals by promoting their translation potential. It has been estimated that over 90% of human solid tumors contain cells with more than two copies of one or more chromosomes [159]; thus these cells have increased folds of transcription rates. If the produced proteins are part of stoichiometric complexes, then the excess of polypeptides not associated to the complexes (or protein machineries) must be removed by UPP degradation. Furthermore, tumorigenesis is marked by oncogenes overexpression, which in general enhances cellular translational capacity, by stimulating the production of ribosomal proteins, the synthesis of ribosomal RNA and the activation of RNA polymerases [160,161]. On the other hand, cancer cells can also promote translation, by inducing the expression of translation initiation factors, eIF3 (eukaryotic translation initiation factor 3) and eIF4F [162]. Therefore, apart from increased oxidative load and metabolic deregulation that impact on proteome integrity and stability, cancer cells are also characterized by enhanced activity of the protein synthesis PN module and the overproduction of mutated polypeptides. Thus, cancer cells are highly dependent on UPP protein degradation in order to adapt to high levels of proteotoxic stress.

## 8. The Ubiquitin-Proteasome liaison in DNA Damaged Responses (DDR)

### 8.1. Ubiquitination and Sumoylation

The importance of ubiquitination in cellular homeodynamics is emphasized by the number of proteins that are modified by ubiquitin molecules and of the factors involved [163]. In addition to ubiquitin, there are several other proteins that are structurally related to ubiquitin and are collectively known as ubiquitin-like proteins (UBLs). UBLs are also attached to target proteins via their C-terminal glycine residues by enzymatic reactions mediated by E1-, E2-, and E3-like ligases. One of the best-characterized UBLs is SUMO (small ubiquitin-related modifier); a small protein of 100 amino acids [164]. Ubiquitination and sumoylation play an essential role in coordinating the function of proteins involved in DSB. Several internal lysine residues (Lys-6, -11, -27, -29, -33, -48 and -63) can be used for the targeting of a protein in a specific pathway and the type of ubiquitination strongly affects the fate of proteins. For instance proteins modified with Lys-48-, Lys-29- and Lys-11- polyubiquitin chains are targeted for degradation via the 26S proteasome, while Lys-63-linked ubiquitin chains are mainly used as a transduction signal that modulates protein functions [165]. Several proteins (more than 250), including DDR involved proteins, are able to recognize ubiquitin and SUMO signals through ubiquitin binding domains (UBDs). Either proteolytic or non-proteolytic ubiquitination have an important regulatory role in DSB signaling and repair [166,167]. The RING finger proteins RNF8/RNF168 are the two most extensively studied RING-type E3 ligases in the DDR pathway [168,169]. Upon phosphorylation of H_2_AX, MDC1 is recruited to DSBs, which then recruits RNF8. RNF8 in association with RNF168 and the E2 enzyme UBC1 (Ubiquitin conjugating enzyme 1) catalyzes the formation of Lys-63-ubiquitin conjugates on H_2_A and H_2_AX. This modification allows the recruitment of several DSB repair factors (including BRCA1 and 53BP1) to the break sites and the suppression of transcriptional events close to the DSBs zone. BRCA1 is thought to promote homologous recombination, while 53BP1 is an important mediator of NHEJ events; however, the antagonistic mechanisms of these proteins are still unclear [170].

As mentioned, sumoylation is another PTM that promotes DSB-associated histone ubiquitination and regulates DSB repair pathway functionality at several levels. There are three SUMOs (SUMO1-3) in mammalian cells. SUMO-2 and SUMO-3 are highly identical except for three residues, while SUMO-1 shares only ~48% sequence identity with SUMO-2 [171]. SUMO isoforms physically accumulate at DSB-modified chromatin and several components of the DDR machinery, such as MDC1, 53BP1, BRCA1, RNF168, HERC2, RAP80 undergo sumoylation. These reactions are performed mainly by SUMO E3, PIAS1 (protein inhibitor of activated STAT1) and PIAS4 (protein inhibitor of activated STAT4) proteins. Depletion of PIAS4 results in loss of RNF168, Lys-63-ubiquitin, 53BP1 and BRCA1 recruitment at DSB sites, while PIAS1 depletion only prevents RAP80 and BRCA1 accumulation [171]. Several studies suggest an extensive crosstalk between sumoylation and ubiquitination. An example of this interaction is the RNF4 factor, which contains SUMO-interacting motifs (SIMs) to facilitate its binding to the sumoylated substrates and promote their turnover through Lys-48-linked ubiquitination [172]. The role of ubiquitination and sumoylation is extended also to the other types of DDR, such BER, NER and normal DNA replication [168,173,174]. Overall, these PTMs are extremely important in facing DNA damage and repair processes.

### 8.2. Proteasomal Degradation of DDR Factors

Proteasome localizes in both the cytosol and in the nucleus where it mostly concentrates in euchromatin, at the periphery of heterochromatin and nucleoli [175]. Proteolytic digestion of key DDR proteins is highly regulated and can occur in a timely fashion [176]. Several proteasome subunits are targets of ATM/ATR kinases mediated phosphorylation, emphasizing the role of proteasome activity at DNA damage responses [29], however, the mechanistic details of how proteasomal degradation is involved in chromatin metabolism and, even more, in DSBs remains to be clarified. DSBs induce rapid polyubiquitination of DDR proteins and therefore topical accumulation of Lys-48-linked ubiquitin chains and proteasome subunits. The proteins to be targeted for proteasomal degradation (e.g., via Lys-48-ubiquitination) are extracted from the chromatin by the ubiquitin-selective segregase p97/VCP [133] (see also above). p97/VCP is recruited by the adaptor complex UFD1–NPL4 (ubiquitin fusion degradation protein 1-nuclear protein localization protein 4) and on RNF8–RNF168 ubiquitin chains [133]. Disruption of p97/VCP leads to accumulation of polyubiquitin chains and impaired recruitment of repair factors [170]. Another target of proteasome degradation and a key component of DDR is MDC1. RFN4 mediates the turnover of MDC1 after Lys-48-ubiquitination [172]. Inhibition of MDC1 through proteasomal degradation suppresses the recruitment of BRCA1 at the sites of DSBs [177], suggesting the importance of proteasomal degradation in the continuity of the DDR cascade. Degradation of BRCA1 is also mediated by the proteasome after HERC2 (HECT-type E3 ligase) targeting; BRCA1, together with its partner BARD1 (BRCA1-associated RING domain 1), have an important role in HR and loss of any of these proteins results in susceptibility to breast cancer [178].

Moreover, CtIP, a factor that enables DNA-end resection, is controlled by multiple protein-protein interactions and PTMs; for instance, cullin3 E3 ligase substrate adaptor Kelch-like protein 15 (KLHL15) promotes CtIP protein turnover via the ubiquitin-proteasome pathway [179]. Another very important factor in DDR responses (that is controlled by proteasomal degradation) is the p53 protein. p53 is considered the “guardian of the genome” because of its broad range of genome protective functions [180]. Over ten ubiquitin E3 ligases have been linked to p53 regulation, but the most studied is the RING E3-type, MDM2/HDM2 ligase [181]. MDM2 binds to the N-terminal of p53, targeting the protein for ubiquitination and subsequent proteasome dependent degradation. The MDM2-p53 feedback loop is highly conserved and enables the p53-mediated checkpoint surveillance, preventing unnecessary cell cycle arrest or cell death [182]. The amazing coordinative role of p53 in stabilizing and protecting the genome is emphasized by its inactivation in the majority of cancers [183]. Finally, proteasome inhibition in human cells (e.g., by the MG132 inhibitor) suppresses the recruitment of DDR factors and the HR pathway, but does not significantly affect the NHEJ pathway [184]. UPP is also involved in the degradation of the MMR pathway components. Specifically, proteasome mediates the degradation of the hMutSα complex [185]. Reduced levels of hMutSα in vitro limit the MMR activity, emphasizing the proteolytic regulatory role of UPP in the MMR pathway [185]. In addition, Exonuclease I is polyubiquitinated and degraded by the proteasome when DNA replication is inhibited during the S phase [186]. The components of the BER pathway, also, require a quick turnover of the proteins involved. Specifically, turnover of BER proteins is supported by two E3 ubiquitin ligases; namely, the Mule/ARF-BP1, which adds an ubiquitin molecule to the BER proteins, and CHIP, which extends the ubiquitin chain and labels proteins for proteasomal degradation [187]. The XRCC1 factor, which directs the assembly of BER complexes at sites of DNA damage, is also a target for degradation by the CHIP E3 ligase [188]. Phosphorylation of XRCC1 by casein kinase 2 (CK2) inhibits its ubiquitination, enhancing XRCC1 stability and allowing thus an efficient BER process [189]. Moreover, the other key component of BER, PARP1 is polyubiquitinated by SUMO-1 and SUMO-3 in both in vitro and in vivo systems [190,191]; notably, proteasomal inhibition is necessary for the polyubiquitination of PARP1, suggesting that it is a target of the UPP mediated degradation [190]. Taken together these findings suggest that UPP is functionally involved in several steps of DDR and of genome repairing processes.

## 9. Autophagy-Lysosome Pathway (ALP) in Genome Integrity

Autophagy is an evolutionarily conserved catabolic process of misfolded proteins, damaged proteins and organelles. In addition, autophagy reduces ROS levels through the recycling of damaged mitochondria, (via an autophagic process known as mitophagy), and the activation of antioxidant mechanisms [192,193]. Constitutive, low level of basal autophagy in normal tissues provides an important homeostatic housekeeping function [194]. Thus, autophagy (as DDR) is essential for cellular and organismal homeostasis. Reportedly, autophagy is activated by DNA damage and is required for several functional outcomes of DDR signaling, including repair of DNA lesions, senescence, cell death and cytokine secretion [195].

Autophagy starts by the formation of the ULK1 complex, which is composed of ULK1 (unc-51-like kinase 1 protein), ATG13, mTOR kinase and RB1CC1 (RB1-inducibile coiled-coil 1). Autophagy activating stimuli inhibit mTOR that under physiological conditions phosphorylates and inhibits the ULK1 and ATG13 proteins of the complex [196]. Phagophore nucleation requires the formation of a complex consisting of the vacuolar protein sorting (VPS) 34, VPS15, Beclin1 and the activating autophagy/beclin-1 regulator 1 (AMBRA1); in this process, B-cell lymphoma 2 (Bcl-2) inhibits autophagy by binding Beclin1, while BCL2-homology 3 (BH3-only) activates the VPS34 complex after displacement of the Bcl-2 protein. The phagophore expands after conjugation of ATG12 to ATG5 that interacts with ATG16 forming the ATG16L complex, which then conjugates phophatidyethanolamine (PE) to microtubule-associated protein 1 light chain 3 (LC3) until the generation of the LC3 II receptor. The expansion of the phagophore continues until the edges surround the cargo, fuse, and form the autophagosome; finally, the autophagosome fuses with lysosomes and its content is being degraded [197].

Several studies have attributed to autophagy an important role in preserving genome integrity. The first observation was reported on mammary epithelial autophagy deficient tumor cells, in which allelic loss of *beclin1* resulted in sensitization to metabolic stress and increased ROS levels [198]. These authors reported that autophagy defects also activate DDR in vitro and in mammary tumors in vivo, promoting gene amplification and tumorigenesis [198]. Loss of autophagy in murine *atg7^−/−^* keratinocytes results in severely increased DNA damage and senescence [199]. Furthermore, reduced autophagy can lead to insufficient ATP production, damaged mitochondria and excessive ROS levels [200], compromising the cell ability to adapt to metabolic stress [201]. Increased ROS generate a vicious pro-oxidative cycle, since ROS by-products can affect the genome and proteome integrity and uncouple the respiratory chain, leading to even more ROS production [202]. In addition, autophagy likely cross talks with the Nrf2 antioxidant responses pathway since, as mentioned above, p62/SQSTM1 dependent degradation of Keap1 leads to Nrf2 release; its translocation into the nucleus, and to activation of antioxidant mechanisms [186]. In support, during stress conditions, autophagy-defective tumor cells accumulate p62/SQSTM1, which in association with ROS further increases cellular damage [203]. Therefore, autophagy is a crucial factor in genome integrity and stability due to suppression of high levels of genotoxic ROS.

Autophagy also plays a key role in nuclear homeodynamics. Several findings have revealed that autophagy is implicated on the turnover of nuclear components, such as micronuclei and chromatin fragments, which is a tightly controlled and selective process. In mammalian cells micronuclei that are positive for the DNA damage marker γH_2_AX are targeted for autophagy dependent degradation by the receptor protein p62/SQSTM1 [204]. Furthermore, chromatin fragments are targeted to autophagic degradation following the induction of oncogene-induced senescence and replicative senescence [205,206]. Dou et al. [207] found that autophagy facilitates oncogene-induced senescence by degrading the nuclear lamina constituent, Lamin B1, and the associated heterochromatin domains LADs (lamin-associated domains). This degradation event occurs preferentially in response to oncogenic transformation, oxidative stress and DNA damage (but not starvation) as a result of nuclear accumulation of lamin B1 regions and its direct interaction with LC3 [207].

Reportedly, autophagy also plays a crucial role during development. Autophagy deficient mice (*beclin1^−/−^*) die early in embryogenesis [208] and *atg5^−/−^* mice are vulnerable to starvation and die perinatally. Autophagy is also extremely important during development and starvation by recycling cytoplasm and macromolecules [201] and thus preserving energy homeostasis [209]. Cells with impaired autophagy (*beclin1*^+/*−*^ and *atg5^−/−^*) are more susceptible to metabolic stress in vitro and are also characterized by increased DNA damage responses [210]. In addition, neonate *atg5^−/−^* mice display high energy depletion, reduced plasma amino acid levels and heart dysfunction [209]. Considering that ATP is necessary for all cellular processes (including DNA replication and DDR) inhibition of autophagy could be likely associated to stalled replication forks [200].

Notably, autophagy seems to also have a more direct role in DNA damage repair processes. Inhibition of histone deacetylases (HDACs) with valporic acid in yeast, results in increased autophagic degradation of the DNA endonuclease Sae2 [211]. Also, cell treatment with rapamycin (an autophagy activator), results in reduced levels of Sae2, suggesting that the acetylation and subsequent degradation of Sae2 through autophagy is likely a new mechanism that connects DNA repair and autophagy. Although according to Robert et al. [211] activation of autophagy can lead to impairment of DDR, several other studies propose that autophagy inactivation reduce the DNA damage repair capacity [212,213,214,215,216]; e.g., Liu et al. [213] showed that *atg7* knockdown leads to impaired DSB repair through the HR pathway. Cells lacking *atg7* are very dependent on NHEJ for DSB repair, and inhibition of NHEJ causes rapid cell death. Furthermore, inhibition of autophagy by genetic knockout of the 200-kDa FAK-family-interacting protein (FIP200; an ULK1-interacting protein that is essential for autophagosome formation) suppressed DNA damage repair and decreased cell viability following IR and treatment with camptothecin; also, knockdown of p62/SQSTM1 alleviated the induced defects in repair and increased cell survival [214]. Recent findings suggest that increased levels of p62/SQSTM1 due to autophagy inhibition could be responsible for reduced DDR; these effects on DNA repair are likely promoted by p62/SQSTM1 that is localized in the nucleus [215,216]. Also, Wang et al. [215] showed that increased levels of p62/SQSTM1 due to inhibition of autophagy reduce DNA damage-induced chromatin ubiquitination. Mechanistically this phenotype is explained by the interaction of p62/SQSTM1 with RNF168. This interaction inhibits RNF168, resulting in impaired chromatin ubiquitination and reduced recruitment of DNA repair proteins, while NHEJ remains unaffected. In addition, Hewitt et al. [216] suggested an alternative mechanism for p62/SQSTM1-depedent autophagy in modulation of DNA repair, as in this study it was shown that p62/SQSTM1 inhibits HR-directed DSB repair through proteasomal degradation of filamin A (FLNA) and RAD51 recombinase in the nucleus. Finally, it was suggested that inhibition of DNA repair mediated by p62/SQSTM1 accelerates ageing. This process can be reversed by caloric restriction, a potent activator of autophagy [216].

Nevertheless, and despite the fact that several studies have associated autophagy to DDR and genome instability, the mechanistic details that underlie the cross talk of these processes remain largely unclear. A systemic study on how these molecular processes are intertwined will provide new concepts for the development of novel therapeutics against ageing and the various age-related pathologies where these pathways are functionally involved.

## 10. Concluding Remarks

**Figure 2:**
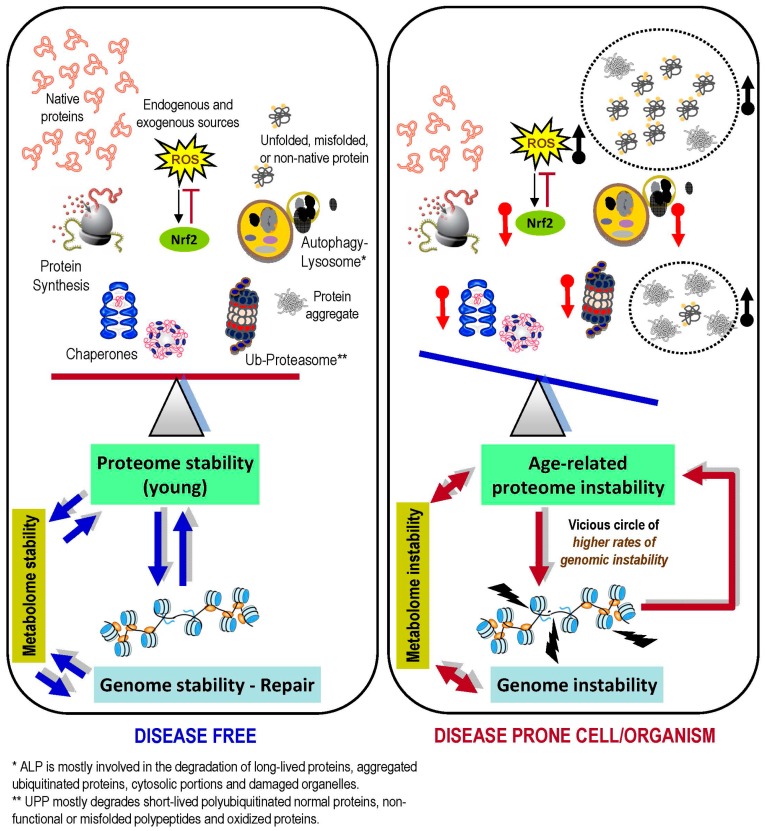
Loss of proteome stability during ageing impacts on genome (and metabolome) integrity, resulting in disease prone cells/organisms. Young biological systems are characterized by low levels of damaged biomolecules due to highly active proteostatic pathways and stress responses (e.g., the Nrf2 pathway). This period of life is characterized by genome stability as a result of precise duplication and effective repair pathways (blue arrows in **left** panel denote balanced cross-talk among shown pathways and regulatory modules). On the other hand, the age-related collapse of proteostatic modules functionality and/or expression levels (red arrows; **upper right** panel), along with increased oxidative stress and the accumulation of non-functional polypeptides and/or protein aggregates (black arrows; **upper right** panel), compromises proteome integrity leading to significantly reduced chances of survival/health due to metabolic alterations and/or genomic instability (caused by ineffective DNA maintenance and/or repair). Eventually, a vicious circle (red arrows; **lower right** panel) may be formed where a mildly unstable genome accelerates proteome instability (and consequently metabolic alterations) due to synthesis of mutated polypeptides, which progressively increase the attrition of protein machines. This vicious circle gradually results in an increasingly stressful cellular landscape that favors the appearance of age-related diseases (e.g., cancer).

It is becoming clear that the role of PN is not solely limited to maintaining proteome stability, but rather PN is coordinating cellular functionality and viability as a whole. Protein machineries assemble and operate in all cellular compartments (e.g., ER, mitochondria, cytosol and nucleus) and also in the inter-/extra-cellular space in order to orchestrate the interactions between cells and to maintain homeodynamics of biological liquids; thus the PN modules face different environmental challenges. Proteome stability and PN functionality declines during ageing [102] or due to constant exposure to stressors [217], leading to accumulation of unfolded/misfolded proteins and of protein aggregates. This end point causes proteome instability that is characterized by increased proteotoxic stress and underlies many pathologies including cancer. As discussed herein, genome stability (e.g., processes like DNA replication and DDR) is continuously followed and coordinated by the curating activity of PN. Considering that aged tissues have deteriorated PN functionality, but they still need to adapt to metabolic and environmental changes, we can hypothesize that increased levels of genome instability in aged cells/tissues is largely a secondary effect of age-related upregulation of proteotoxic stress and low efficiency of PN functionality (Figure 2). Nevertheless, as this is a relatively new concept, further studies are needed in order to address if loss of proteostasis is the triggering event of increased genome instability; or if the latter (apart from PN dysfunction) also associates with stochastic accumulation of genetic mutations. As most of the performed studies analyze single components of the PN we are currently focusing on a more global/systemic view of PN alterations during ageing and/or cancer (an age-related disease characterized by immense genomic instability) in order to address the aforementioned question. Finally, considering that specific inhibition of PN modules (e.g., proteasome) have demonstrated clinical efficacy in the treatment of hematological cancers [218], the identification of key PN modules in the preservation of genome integrity will likely provide novel strategies and pharmacological targets for preventing and/or treating advanced tumors.

## Abbreviations

53BP1	p53-binding protein 1
ALP	autophagy-lysosome pathway
AMPK	adenosine monophosphate-activated protein kinase
AP	apurinic/apyrimidinic sites
ATF	activating transcription factor
ATM	ataxia telangiectasia mutated
ATR	ataxia telangiectasia mutated and Rad3 related
ATRIP	ataxia telangiectasia mutated and Rad3 related interacting protein
BER	base-excision repair
BRCA1	breast cancer type 1 susceptibility protein
CDK	cyclin-dependent kinase
CHEK1	checkpoint kinase-1
CHEK2	checkpoint kinase-2
DDR	DNA damage response
DNA-PKcs	DNA-dependent protein kinase catalytic subunit
DSB	double strand break
ER	endoplasmic reticulum
ERAD	endoplasmic reticulum-associated degradation
FA	Fanconi anemia complex
FIP200	200-kDa FAK-family-interacting protein
FLNA	filamin A
FoxO	forkhead box O
GPX1	glutathione peroxidase 1
GSTD1	glutathione *S*-transferase 1
HDACs	histone deacetylases
HR	homologous recombination
HIF-1	hypoxia-inducible factor 1
Hsf1	heat shock factor 1
Hsp	heat shock protein
IRE1	inositol-requiring enzyme 1
IR	ionizing radiation
Keap1	kelch-like ECH-associated protein 1
KLHL15	kelch-like protein 15
LAD	lamin-associated domain
maf	v-Maf avian musculoaponeurotic fibrosarcoma oncogene homolog
MDC1	mediator of DNA damage checkpoint 1
MDM2	mouse double minute 2 homolog
MMR	mismatch repair
MRN	MRE11/RAD50/NBS1 complex
NQO1	NA(D)PH quonine dehydrogenase 1
NER	nucleotide excision repair
NHEJ	non-homologous end joining
Nrf2	nuclear factor erythroid 2-related factor 2
PERK	protein kinase R-like endoplasmic reticulum kinase
PIAS	protein inhibitor of activated STAT
PICHROS	protein-induced chromatin stress
PIKK	phosphatidylinositol 3-kinase-related kinase
PKR	protein kinase R
PN	proteostasis network
PTM	post-translational modification
RB1CC1	RB1-inducible coiled-coil 1
RNF168	ring finger protein 168
ROS	reactive oxygen species
SIRT1	sirtuin 1
sHSP	small heat-shock protein
SOD	superoxide dismutase
SSB	single strand brake
TIGAR	TP53-inducible glycolysis and apoptosis regulator
TOR	target of rapamycin
UBD	ubiquitin binding domain
UBL	ubiquitin-like protein
ULK-1	unc-51 like autophagy activating kinase 1
UPP	ubiquitin-proteasome pathway
UPR	unfolded protein response
UPRER	endoplasmic reticulum unfolded protein response
UV	ultra violet
VCP	valosin-containing protein
XBP1	X box-binding protein

## References

[1] Lord C.J., Ashworth A. (2012). The DNA damage response and cancer therapy. Nature.

[2] Jackson S.P., Bartek J. (2009). The DNA-damage response in human biology and disease. Nature.

[3] Tsakiri E.N., Trougakos I.P. (2015). The amazing ubiquitin-proteasome system: Structural components and implication in aging. Int. Rev. Cell Mol. Biol..

[4] Vilchez D., Saez I., Dillin A. (2014). The role of protein clearance mechanisms in organismal ageing and age-related diseases. Nat. Commun..

[5] Akerfelt M., Morimoto R.I., Sistonen L. (2010). Heat shock factors: Integrators of cell stress, development and lifespan. Nat. Rev. Mol. Cell. Biol..

[6] Arnold J., Grune T. (2002). PARP-mediated proteasome activation: A co-ordination of DNA repair and protein degradation?. Bioessays.

[7] Morimoto R.I., Cuervo A.M. (2014). Proteostasis and the aging proteome in health and disease. J. Gerontol. Ser. A.

[8] Pearl L.H., Schierz A.C., Ward S.E., Al-Lazikani B., Pearl F.M. (2015). Therapeutic opportunities within the DNA damage response. Nat. Rev. Cancer.

[9] Yang X.J., Seto E. (2007). HATs and HDACs: From structure, function and regulation to novel strategies for therapy and prevention. Oncogene.

[10] Tarrant M.K., Cole P.A. (2009). The chemical biology of protein phosphorylation. Annu. Rev. Biochem..

[11] Welchman R.L., Gordon C., Mayer R.J. (2005). Ubiquitin and ubiquitin-like proteins as multifunctional signals. Nat. Rev. Mol. Cell Biol..

[12] Zharkov D.O. (2008). Base excision DNA repair. Cell. Mol. Life Sci..

[13] Kolodner R.D., Marsischky G.T. (1999). Eukaryotic DNA mismatch repair. Curr. Opin. Genet. Dev..

[14] Kunkel T.A., Erie D.A. (2005). DNA mismatch repair. Annu. Rev. Biochem..

[15] Plotz G., Raedle J., Brieger A., Trojan J., Zeuzem S. (2002). hMutSα forms an ATP-dependent complex with hMutLα and hMutLβ on DNA. Nucleic Acids Res..

[16] Li G.M., Modrich P. (1995). Restoration of mismatch repair to nuclear extracts of H6 colorectal tumor cells by a heterodimer of human MutL homologs. Proc. Natl. Acad. Sci. USA.

[17] Li G.M. (2008). Mechanisms and functions of DNA mismatch repair. Cell Res..

[18] Hanawalt A., Spivak G. (2008). Transcription-coupled DNA repair: Two decades of progress and surprises. Nat. Rev. Mol. Cell Biol..

[19] Spivak G. (2015). Nucleotide excision repair in humans. DNA Repair.

[20] Sugasawa K. (2016). Molecular mechanisms of DNA damage recognition for mammalian nucleotide excision repair. DNA Repair.

[21] Chapman J.R., Taylor M.R., Boulton S.J. (2012). Playing the end game: DNA double-strand break repair pathway choice. Mol. Cell.

[22] Zha S., Guo C., Boboila C., Oksenych V., Cheng H.L., Zhang Y., Wesemann D.R., Yuen G., Patel H., Goff P.H. (2011). ATM damage response and XLF repair factor are functionally redundant in joining DNA breaks. Nature.

[23] Hiom K. (2010). Coping with DNA double strand breaks. DNA Repair.

[24] McVey M., Lee S.E. (2008). MMEJ repair of double-strand breaks (director’s cut): Deleted sequences and alternative endings. Trends Genet..

[25] Kozlov S.V., Graham M.E., Jakob B., Tobias F., Kijas A.W., Tanuji M., Chen P., Robinson P.J., Taucher-Scholz G., Suzuki K. (2011). Autophosphorylation and ATM activation: Additional sites add to the complexity. J. Biol. Chem..

[26] Chowdhury D., Xu X., Zhong X., Ahmed F., Zhong J., Liao J., Dykxhoorn D.M., Weinstock D.M., Pfeifer G.P., Lieberman J. (2008). A PP4-phosphatase complex dephosphorylates γ-H_2_AX generated during DNA replication. Mol. Cell.

[27] Ohashi E., Takeishi Y., Ueda S., Tsurimoto T. (2014). Interaction between Rad9-Hus1-Rad1 and TopBP1 activates ATR-ATRIP and promotes TopBP1 recruitment to sites of UV-damage. DNA Repair.

[28] Sideridou M., Zakopoulou R., Evangelou K., Liontos M., Kotsinas A., Rampakakis E., Gagos S., Kahata K., Grabusic K., Gkouskou K. (2011). Cdc6 expression represses E-cadherin transcription and activates adjacent replication origins. J. Cell Biol..

[29] Matsuoka S., Ballif B.A., Smogorzewska A., McDonald E.R., Hurov K.E., Luo J., Bakalarski C.E., Zhao Z., Solimini N., Lerenthal Y. (2007). ATM and ATR substrate analysis reveals extensive protein networks responsive to DNA damage. Science.

[30] Bennetzen M.V., Larsen D.H., Bunkenborg J., Bartek J., Lukas J., Andersen J.S. (2010). Site-specific phosphorylation dynamics of the nuclear proteome during the DNA damage response. Mol. Cell. Proteom..

[31] Stucki M., Clapperton J.A., Mohammad D., Yaffe M.B., Smerdon S.J., Jackson S.P. (2005). MDC1 directly binds phosphorylated histone H2AX to regulate cellular responses to DNA double-strand breaks. Cell.

[32] Stewart G.S., Wang B., Bignell C.R., Taylor A.M., Elledge S.J. (2003). MDC1 is a mediator of the mammalian DNA damage checkpoint. Nature.

[33] Bartek J., Lukas J. (2007). DNA damage checkpoints: From initiation to recovery or adaptation. Curr. Opin. Cell Biol..

[34] Schwarz J.K., Lovly C.M., Piwnica-Worms H. (2003). Regulation of the Chk2 protein kinase by oligomerization-mediated cis- and trans-phosphorylation. Mol. Cancer Res..

[35] Velimezi G., Liontos M., Vougas K., Roumeliotis T., Bartkova J., Sideridou M., Dereli-Oz A., Kocylowski M., Pateras I.S., Evangelou K. (2013). Functional interplay between the DNA-damage-response kinase ATM and ARF tumour suppressor protein in human cancer. Nat. Cell Biol..

[36] Olsson A., Manzl C., Strasser A., Villunger A. (2007). How important are post-translational modifications in p53 for selectivity in target-gene transcription and tumour suppression?. Cell Death Differ..

[37] Shinozaki K., Yamaguchi-Shinozaki K., Seki M. (2003). Regulatory network of gene expression in the drought and cold stress responses. Curr. Opin. Plant Biol..

[38] Maya R., Balass M., Kim S.T., Shkedy D., Leal J.F., Shifman O., Moas M., Buschmann T., Ronai Z., Shiloh Y. (2001). ATM-dependent phosphorylation of Mdm2 on serine 395: Role in p53 activation by DNA damage. Genes Dev..

[39] Roos W.P., Thomas A.D., Kaina B. (2016). DNA damage and the balance between survival and death in cancer biology. Nat. Rev. Cancer.

[40] Papamichos-Chronakis M., Peterson C.L. (2013). Chromatin and the genome integrity network. Nat. Rev. Genet..

[41] Negrini S., Gorgoulis V.G., Halazonetis T.D. (2010). Genomic instability—An evolving hallmark of cancer. Nat. Rev. Mol. Cell Biol..

[42] Hanahan D., Weinberg R.A. (2011). Hallmarks of cancer: The next generation. Cell.

[43] Trougakos I.P., Sesti F., Tsakiri E., Gorgoulis V.G. (2013). Non-enzymatic post-translational protein modifications and proteostasis network deregulation in carcinogenesis. J. Proteom..

[44] Powers E.T., Morimoto R.I., Dillin A., Kelly J.W., Balch W.E. (2009). Biological and chemical approaches to diseases of proteostasis deficiency. Annu. Rev. Biochem..

[45] Fribley A., Zhang K., Kaufman R.J. (2009). Regulation of apoptosis by the unfolded protein response. Methods Mol. Biol..

[46] Nakka V.P., Prakash-Babu P., Vemuganti R. (2016). Crosstalk between endoplasmic reticulum stress, oxidative stress, and autophagy: Potential therapeutic targets for acute CNS injuries. Mol. Neurobiol..

[47] Niforou K., Cheimonidou C., Trougakos I.P. (2014). Molecular chaperones and proteostasis regulation during redox imbalance. Redox Biol..

[48] Kim Y.E., Hipp M.S., Bracher A., Hayer-Hartl M., Hartl F.U. (2013). Molecular chaperone functions in protein folding and proteostasis. Annu. Rev. Biochem..

[49] Davies K.J. (2001). Degradation of oxidized proteins by the 20S proteasome. Biochimie.

[50] Grune T., Merker K., Sandig G., Davies K.J. (2003). Selective degradation of oxidatively modified protein substrates by the proteasome. Biochem. Biophys. Res. Commun..

[51] Saeki Y., Tanaka K. (2012). Assembly and function of the proteasome. Methods Mol. Biol..

[52] Wang H., Song P., Du L., Tian W., Yue W., Liu M., Li D., Wang B., Zhu Y., Cao C. (2011). Parkin ubiquitinates Drp1 for proteasome-dependent degradation: Implication of dysregulated mitochondrial dynamics in Parkinson disease. Biol. Chem..

[53] Wiedemann N., Stiller S.B., Pfanner N. (2013). Activation and degradation of mitofusins: Two pathways regulate mitochondrial fusion by reversible ubiquitylation. Mol. Cell.

[54] Gumeni S., Trougakos I.P. (2016). Cross talk of proteostasis and mitostasis in cellular homeodynamics, ageing, and disease. Oxid. Med. Cell. Longev..

[55] Levine B., Kroemer G. (2008). Autophagy in the pathogenesis of disease. Cell.

[56] Youle R.J., Narendra D.P. (2011). Mechanisms of mitophagy. Nat. Rev. Mol. Cell Biol..

[57] Nedić O., Rattan S.I., Grune T., Trougakos I.P. (2013). Molecular effects of advanced glycation end products on cell signalling pathways, ageing and pathophysiology. Free Radic. Res..

[58] Mizushima N., Levine B., Cuervo A.M., Klionsky D.J. (2008). Autophagy fights disease through cellular self-digestion. Nature.

[59] Cuervo A.M. (2010). Chaperone-mediated autophagy: Selectivity pays off. Trends Endocrinol. Metab..

[60] Wong E., Cuervo A.M. (2010). Autophagy gone awry in neurodegenerative diseases. Nat. Neurosci..

[61] Williams A.B., Schumacher B. (2016). p53 in the DNA-damage-repair process. Cold Spring Harb. Perspect. Med..

[62] Calderwood S.K., Murshid A., Prince T. (2009). The shock of aging: Molecular chaperones and the heat shock response in longevity and aging—A mini-review. Gerontology.

[63] Van der Horst A., Burgering B.M. (2007). Stressing the role of FoxO proteins in lifespan and disease. Nat. Rev. Mol. Cell Biol..

[64] Sykiotis G.P., Bohmann D. (2010). Stress-activated cap‘n’collar transcription factors in aging and human disease. Sci. Signal..

[65] Tsakiri E.N., Sykiotis G.P., Papassideri I.S., Terpos E., Dimopoulos M.A., Gorgoulis V.G., Bohmann D., Trougakos I.P. (2013). Proteasome dysfunction in Drosophila signals to an Nrf2-dependent regulatory circuit aiming to restore proteostasis and prevent premature aging. Aging Cell.

[66] Morimoto R.I., Driessen A.J., Hegde R.S., Langer T. (2011). The life of proteins: The good, the mostly good and the ugly. Nat. Struct. Mol. Biol..

[67] Labbadia J., Morimoto R.I. (2015). Repression of the heat shock response is a programmed event at the onset of reproduction. Mol. Cell.

[68] Ron D., Walter P. (2007). Signal integration in the endoplasmic reticulum unfolded protein response. Nat. Rev. Mol. Cell Biol..

[69] Rutkowski D.T., Kaufman R.J. (2007). That which does not kill me makes me stronger: Adapting to chronic ER stress. Trends Biochem. Sci..

[70] Tsai Y.C., Weissman A.M. (2010). The unfolded protein response, degradation from endoplasmic reticulum and cancer. Genes Cancer.

[71] Naidoo N. (2009). Cellular stress/the unfolded protein response: Relevance to sleep and sleep disorders. Sleep Med. Rev..

[72] Yamamori T., Meike S., Nagane M., Yasui H., Inanami O. (2013). ER stress suppresses DNA double-strand break repair and sensitizes tumor cells to ionizing radiation by stimulating proteasomal degradation of Rad51. FEBS Lett..

[73] Bindra R.S., Schaffer P.J., Meng A., Woo J., Måseide K., Roth M.E., Lizardi P., Hedley D.W., Bristow R.G., Glazer P.M. (2004). Down-regulation of Rad51 and decreased homologous recombination in hypoxic cancer cells. Mol. Cell. Biol..

[74] Li X., Zhang K., Li Z. (2011). Unfolded protein response in cancer: The physician’s perspective. J. Hematol. Oncol..

[75] Oommen D., Prise K.M. (2013). Down-regulation of PERK enhances resistance to ionizing radiation. Biochem. Biophys. Res. Commun..

[76] Raven J.F., Baltzis D., Wang S., Mounir Z., Papadakis A.I., Gao H.Q., Koromilas A.E. (2008). PKR and PKR-like endoplasmic reticulum kinase induce the proteasome-dependent degradation of cyclin D1 via a mechanism requiring eukaryotic initiation factor 2α phosphorylation. J. Biol. Chem..

[77] Fu M., Wang C., Li Z., Sakamaki T., Pestell R.G. (2004). Minireview: Cyclin D1: Normal and abnormal functions. Endocrinology.

[78] Shimura T., Kakuda S., Ochiai Y., Nakagawa H., Kuwahara Y., Takai Y., Kobayashi J., Komatsu K., Fukumoto M. (2010). Acquired radioresistance of human tumor cells by DNA-PK/AKT/GSK3β-mediated cyclin D1 overexpression. Oncogene.

[79] Lee A.S. (2001). The glucose-regulated proteins: Stress induction and clinical applications. Trends Biochem. Sci..

[80] Little E., Ramakrishnan M., Roy B., Gazit G., Lee A.S. (1994). The glucose-regulated proteins (GRP78 and GRP94): Functions, gene regulation, and applications. Crit. Rev. Eukaryot. Gene Expr..

[81] Rao R.V., Castro-Obregon S., Frankowski H., Schuler M., Stoka V., del Rio G., Bredesen D.E., Ellerby H.M. (2002). Coupling endoplasmic reticulum stress to the cell death program. An Apaf-1-independent intrinsic pathway. J. Biol. Chem..

[82] Reddy R.K., Mao C., Baumeister P., Austin R.C., Kaufman R.J., Lee A.S. (2003). Endoplasmic reticulum chaperone protein GRP78 protects cells from apoptosis induced by topoisomerase inhibitors: Role of ATP binding site in suppression of caspase-7 activation. J. Biol. Chem..

[83] Baumeister P., Luo S., Skarnes W.C., Sui G., Seto E., Shi Y., Lee A.S. (2005). Endoplasmic reticulum stress induction of the Grp78/BiP promoter: Activating mechanisms mediated by YY1 and its interactive chromatin modifiers. Mol. Cell. Biol..

[84] Boisvert F.M., Dery U., Masson J.Y., Richard S. (2005). Arginine methylation of MRE11 by PRMT1 is required for DNA damage checkpoint control. Genes Dev..

[85] Groenendyk J., Agellon L.B., Michalak M. (2013). Coping with endoplasmic reticulum stress in the cardiovascular system. Annu. Rev. Physiol..

[86] Kasper L.H., Boussouar F., Boyd K., Xu W., Biesen M., Rehg J., Baudino T.A., Cleveland J.L., Brindle P.K. (2005). Two transactivation mechanisms cooperate for the bulk of HIF-1-responsive gene expression. EMBO J..

[87] Jung J.E., Lee H.G., ChoI H., Chung D.H., Yoon S.H., Yang Y.M., Lee J.W., Choi S., Park J.W., Ye S.K. (2005). STAT3 is a potential modulator of HIF-1-mediated VEGF expression in human renal carcinoma cells. FASEB J..

[88] Islam K.N., Mendelson C.R. (2006). Permissive effects of oxygen on cyclic AMP and interleukin1 stimulation of surfactant protein A gene expression are mediated by epigenetic mechanisms. Mol. Cell. Biol..

[89] Johnson A.B., Barton M.C. (2007). Hypoxia-induced and stress-specific changes in chromatin structure and function. Mutat. Res..

[90] Bristow R.G., Hill R.P. (2008). Hypoxia and metabolism: Hypoxia, DNA repair and genetic instability. Nat. Rev. Cancer.

[91] Valko M., Rhodes C.J., Moncol J., Izakovic M., Mazur M. (2006). Free radicals, metals and antioxidants in oxidative stress-induced cancer. Chem. Biol. Interact..

[92] Wang M.Y., Dhingra K., Hittelman W.N., Liehr J.G., deAndrade M., Li D.H. (1996). Lipid peroxidation-induced putative malondialdehyde–DNA adducts in human breast tissues. Cancer Epidemiol. Biomark. Prev..

[93] Boonstra J., Post J.A. (2004). Molecular events associated with reactive oxygen species and cell cycle progression in mammalian cells. Gene.

[94] Schafer F.Q., Buettner G.R. (2001). Redox environment of the cell as viewed through the redox state of the glutathione disulfide/glutathione couple. Free Radic. Biol Med..

[95] Reuter S., Gupta S.C., Chaturvedi M.M., Aggarwal B.B. (2010). Oxidative stress, inflammation, and cancer: How are they linked?. Free Radic. Biol. Med..

[96] Motohashi H., Yamamoto M. (2004). Nrf2-Keap1 defines a physiologically important stress response mechanism. Trends Mol. Med..

[97] Rada P., Rojo A.I., Chowdhry S., McMahon M., Hayes J.D., Cuadrado A. (2011). SCF/β-TrCP promotes glycogen synthase kinase 3-dependent degradation of the Nrf2 transcription factor in a Keap1-independent manner. Mol. Cell. Biol..

[98] Taguchi K., Motohashi H., Yamamoto M. (2011). Molecular mechanisms of the Keap1–Nrf2 pathway in stress response and cancer evolution. Genes Cells.

[99] McMahon M., Itoh K., Yamamoto M., Hayes J.D. (2003). Keap1-dependent proteasomal degradation of transcription factor Nrf2 contributes to the negative regulation of antioxidant response element-driven gene expression. J. Biol. Chem..

[100] Zhang D.D., Hannink M. (2003). Distinct cysteine residues in Keap1 are required for Keap1-dependent ubiquitination of Nrf2 and for stabilization of Nrf2 by chemopreventive agents and oxidative stress. Mol. Cell. Biol..

[101] Kwak M.K., Wakabayashi N., Greenlaw J.L., Yamamoto M., Kensler T.W. (2003). Antioxidants enhance mammalian proteasome expression through the Keap1-Nrf2 signalling pathway. Mol. Cell. Biol..

[102] Tsakiri E.N., Sykiotis G.P., Papassideri I.S., Gorgoulis V.G., Bohmann D., Trougakos I.P. (2013). Differential regulation of proteasome functionality in reproductive vs. somatic tissues of Drosophila during aging or oxidative stress. FASEB J..

[103] Friedberg E.C. (2003). DNA damage and repair. Nature.

[104] Ciccia A., Elledge J.S. (2010). The DNA Damage Response: Making it safe to play with knives. Mol. Cell.

[105] Gutowski M., Kowalczyk S. (2013). A study of free radical chemistry: Their role and pathophysiological significance. Acta Biochim. Pol..

[106] Wiseman H., Halliwell B. (1996). Damage to DNA by reactive oxygen and nitrogen species: Role in inflammatory disease and progression to cancer. Biochem. J..

[107] Munoz-Gamez J.A., Rodriguez-Vargas J.M., Quiles-Perez R., Aguilar-Quesada R., Martin-Oliva D., de Murcia G., de Murcia J.M., Almendros A., de Almodóvar M.R., Oliver F.J. (2009). PARP-1 is involved in autophagy induced by DNA damage. Autophagy.

[108] Rodríguez-Vargas J.M., Ruiz-Magaña M.J., Ruiz-Ruiz C., Majuelos-Melguizo J., Peralta-Leal A., Rodríguez M.I., Muñoz-Gámez J.A., de Almodóvar M.R., Siles E., Rivas A.L. (2012). ROS-induced DNA damage and PARP-1 are required for optimal induction of starvation-induced autophagy. Cell Res..

[109] Trachootham D., Alexandre J., Huang P. (2009). Targeting cancer cells by ROS-mediated mechanisms: A radical therapeutic approach?. Nat. Rev. Drug Discov..

[110] Ogrunc M., Di Micco R., Liontos M., Bombardelli L., Mione M., Fumagalli M., Gorgoulis V.G., d’Adda di Fagagna F. (2014). Oncogene-induced reactive oxygen species fuel hyperproliferation and DNA damage response activation. Cell Death Differ..

[111] DeNicola G.M., Karreth F.A., Humpton T.J., Gopinathan A., Wei C., Frese K., Mangal D., Kenneth H.Y., Yeo C.J., Calhoun E.S. (2011). Oncogene-induced Nrf2 transcription promotes ROS detoxification and tumorigenesis. Nature.

[112] Lau A., Wang X.J., Zhao F., Villeneuve N.F., Wu T., Jiang T., Sun Z., White E., Zhang D.D. (2010). Noncanonical Mechanism of Nrf2 Activation by Autophagy Deficiency: Direct Interaction between Keap1 and p62. Mol. Cell. Biol..

[113] Chio I.I., Jafarnejad S.M., Ponz-Sarvise M., Park Y., Rivera K., Palm W., Wilson J., Sangar V., Hao Y., Öhlund D. (2016). NRF2 promotes tumor maintenance by modulating mRNA translation in pancreatic cancer. Cell.

[114] Bensaad K., Tsuruta A., Selak M.A., Vidal M.N., Nakano K., Bartrons R., Gottlieb E., Vousden K.H. (2006). TIGAR, a p53-inducible regulator of glycolysis and apoptosis. Cell.

[115] Cheung E.C., Ludwig R.L., Vousden K.H. (2012). Mitochondrial localization of TIGAR under hypoxia stimulates HK2 and lowers ROS and cell death. Proc. Natl. Acad. Sci. USA.

[116] Kim S.B., Pandita R.K., Eskiocak U., Ly P., Kaisani A., Kumar R., Cornelius C., Wright W.E., Pandita T.K., Shay J.W. (2012). Targeting of Nrf2 induces DNA damage signalling and protects colonic epithelial cells from ionizing radiation. Proc. Natl. Acad. Sci. USA.

[117] Jódar L., Mercken E.M., Ariza J., Younts C., González-Reyes J.A., Alcaín F.J., Burón I., de Cabo R., Villalba J.M. (2011). Genetic deletion of Nrf2 promotes immortalization and decreases life span of murine embryonic fibroblasts. J. Gerontol. Ser. A.

[118] Faraonio R., Vergara P., Di Marzo D., Pierantoni M.G., Napolitano M., Russo T., Cimino F. (2006). p53 suppresses the Nrf2-dependent transcription of antioxidant response genes. J. Biol. Chem..

[119] Walerych D., Lisek K., Sommaggio R., Piazza S., Ciani Y., Dalla E., Rajkowska K., Gaweda-Walerych K., Ingallina E., Tonelli C. (2016). Proteasome machinery is instrumental in a common gain-of-function program of the p53 missense mutants in cancer. Nat. Cell Biol..

[120] Chen W., Sun Z., Wang X.J., Jiang T., Huang Z., Fang D., Zhang D.D. (2009). Direct interaction between Nrf2 and p21(Cip1/WAF1) upregulates the Nrf2-mediated antioxidant response. Mol. Cell.

[121] Galanos P., Vougas K., Walter D., Polyzos A., Maya-Mendoza A., Haagensen E.J., Kokkalis A., Roumelioti F.M., Gagos S., Tzetis M. (2016). Chronic p53-independent p21 expression causes genomic instability by deregulating replication licensing. Nat. Cell Biol..

[122] Kriegenburg F., Jakopec V., Poulsen E.G., Nielsen S.V., Roguev A., Krogan N., Gordon C., Fleig U., Hartmann-Petersen R. (2014). A chaperone-assisted degradation pathway targets kinetochore proteins to ensure genome stability. PLoS Genet..

[123] Tsai Y.L., Chiang Y.R., Narberhaus F., Baron C., Lai E.M. (2010). The small heat-shock protein HspL is a VirB8 chaperone promoting type IV secretion-mediated DNA transfer. J. Biol. Chem..

[124] Bai F., Xi J.H., Wawrousek E.F., Fleming T.P., Andley U.P. (2003). Hyperproliferation and p53 status of lens epithelial cells derived from a B-crystallin knockout mice. J. Biol. Chem..

[125] Hunt C.R., Dix D.J., Sharma G.G., Pandita R.K., Gupta A., Funk M., Pandita T.K. (2004). Genomic instability and enhanced radiosensitivity in Hsp70.1- and Hsp70.3-deficient mice. Mol. Cell. Biol..

[126] Dorard C., de Thonel A., Collura A., Marisa L., Svrcek M., Lagrange A., Jego G., Wanherdrick K., Joly A.L., Buhard O. (2011). Expression of a mutant HSP110 sensitizes colorectal cancer cells to chemotherapy and improves disease prognosis. Nat. Med..

[127] Gao J., Zhu Y., Zhou W., Molinier J., Dong A., Shen W.H. (2012). NAP1 family histone chaperones are required for somatic homologous recombination in Arabidopsis. Plant Cell.

[128] Nystrom T., Yang J., Molin M. (2012). Peroxiredoxins, gerontogenes linking aging to genome instability and cancer. Genes Dev..

[129] Quinlan R.A., Ellis R.J. (2013). Chaperones: Needed for both the good times and the bad times. Philos. Trans. R. Soc. Lond. B.

[130] Vaz B., Halder S., Ramadan K. (2013). Role of p97/VCP (Cdc48) in genome stability. Front. Genet..

[131] Ramadan K., Bruderer R., Spiga F.M., Popp O., Baur T., Gotta M., Meyer H.H. (2007). Cdc48/p97promotes reformation of the nucleus by extracting the kinase Aurora B from chromatin. Nature.

[132] Franz A., Orth M., Pirson P.A., Sonneville R., Blow J.J., Gartner A., Stemmann O., Hoppe T. (2011). CDC48/p97 coordinates CDT1 degradation with GINS chromatin dissociation to ensure faithful DNA replication. Mol. Cell.

[133] Meerang M., Ritz D., Paliwal S., Garajova Z., Bosshard M., Mailand N., Janscak P., Hübscher U., Meyer H., Ramadan K. (2011). The ubiquitin-selective segregase VCP/p97 orchestrates the response to DNA double strand breaks. Nat. Cell Biol..

[134] Raman M., Havens C.G., Walter J.C., Harper J.W. (2011). A genome wide screen identifies p97 as an essential regulator of DNA damage-dependent CDT1destruction. Mol. Cell.

[135] Verma R., Oania R., Fang R., Smith G.T., Deshaies R.J. (2011). Cdc48/p97 mediates UV-dependent turnover of RNAPolII. Mol. Cell.

[136] Mouysset J., Deichsel A., Moser S., Hoege C., Hyman A.A., Gartner A., Hoppe T. (2008). Cell cycle progression requires the CDC48-UFD1/NPL-4 complex for efficient DNA replication. Proc. Natl. Acad. Sci. USA.

[137] Deichsel A., Mouysset J., Hoppe T. (2009). The ubiquitin-selective chaperone CDC48/p97, a new player in DNA replication. Cell Cycle.

[138] Mittelman D., Sykoudis K., Hersh M., Lin Y., Wilson J.H. (2010). Hsp90 modulates CAG repeat instability in human cells. Cell Stress Chaperones.

[139] Noguchi M., Yu D., Hirayama R., Ninomiya Y., Sekine E., Kubota N., Ando K., Okayasu R. (2006). Inhibition of homologous recombination repair in irradiated tumor cells pretreated with Hsp90 inhibitor 17-allylamino-17-demethoxygeldanamycin. Biochem. Biophys. Res. Commun..

[140] Enders G.H. (2008). Expanded roles for Chk1 in genome maintenance. J. Biol. Chem..

[141] Kaplan K.B., Li R. (2012). A prescription for “stress” the role of Hsp90 in genome stability and cellular adaptation. Trends Cell Biol..

[142] Buszczak M., Signer R.A., Morrison S.J. (2014). Cellular differences in protein synthesis regulate tissue homeostasis. Cell.

[143] Hershko A., Ciechanover A. (1998). The ubiquitin system. Annu. Rev. Biochem..

[144] Hochstrasser M. (2009). Origin and function of ubiquitin-like proteins. Nature.

[145] Wiśniewski J.R., Hein M.Y., Cox J., Mann M. (2014). A “proteomic ruler” for protein copy number and concentration estimation without spike-in standards. Mol. Cell. Proteom..

[146] Peth A., Nathan J.A., Goldberg A.L. (2013). The ATP costs and time required to degrade ubiquitinated proteins by the 26 S proteasome. J. Biol. Chem..

[147] Gendron J.M., Webb K., Yang B., Rising L., Zuzow N., Bennett E.J. (2016). Using the ubiquitin-modified proteome to monitor distinct and spatially restricted protein homeostasis dysfunction. Mol. Cell. Proteom..

[148] Harper J.W., Bennett E.J. (2016). Proteome complexity and the forces that drive proteome imbalance. Nature.

[149] Lee M.T., Bonneau A.R., Takacs C.M., Bazzini A.A., DiVito K.R., Fleming E.S., Giraldez A.J. (2013). Nanog, Pou5f1 and SoxB1 activate zygotic gene expression during the maternal-to-zygotic transition. Nature.

[150] Jovanovic M., Rooney M.S., Mertins P., Przybylski D., Chevrier N., Satija R., Rodriguez E.H., Fields A.P., Schwartz S., Raychowdhury R. (2015). Dynamic profiling of the protein life cycle in response to pathogens. Science.

[151] Walter D., Hoffmann S., Komseli E.S., Rappsilber J., Gorgoulis V., Sørensen C.S. (2016). SCF(Cyclin F)-dependent degradation of CDC6 suppresses DNA re-replication. Nat. Commun..

[152] Roseaulin L.C., Noguchi C., Noguchi E. (2013). Proteasome-dependent degradation of replisome components regulates faithful DNA replication. Cell Cycle.

[153] Ullrich O., Reinheckel T., Sitte N., Hass R., Grune T., Davies K.J. (1999). Poly-ADP ribose polymerase activates nuclear proteasome to degrade oxidatively damaged histones. Proc. Natl. Acad. Sci. USA.

[154] Lam Y.W., Lamond A.I., Mann M., Andersen J.S. (2007). Analysis of nucleolar protein dynamics reveals the nuclear degradation of ribosomal proteins. Curr. Biol..

[155] Iwata A., Nagashima Y., Matsumoto L., Suzuki T., Yamanaka T., Date H., Deoka K., Nukina N., Tsuji S. (2009). Intranuclear degradation of polyglutamine aggregates by the ubiquitin-proteasome system. J. Biol. Chem..

[156] McBride W.H., Iwamoto K.S., Syljuasen R., Pervan M., Pajonk F. (2003). The role of theubiquitin/proteasome system in cellular responses to radiation. Oncogene.

[157] Yew P.R. (2001). Ubiquitin-mediated proteolysis of vertebrate G1- and S-phase regulators. J. Cell. Physiol..

[158] Deshaies R.J. (2014). Proteotoxic crisis, the ubiquitin-proteasome system, and cancer therapy. BMC Biol..

[159] Weaver B.A., Cleveland D.W. (2006). Does aneuploidy cause cancer?. Curr. Opin. Cell Biol..

[160] Geiger T., Cox J., Mann M. (2010). Proteomic changes resulting from gene copy number variations in cancer cells. PLoS Genet..

[161] Hsieh A.L., Walton Z.E., Altman B.J., Stine Z.E., Dang C.V. (2015). MYC and metabolism on the path to cancer. Semin. Cell Dev. Biol..

[162] Ruggero D. (2013). Translational control in cancer etiology. Cold Spring Harb. Perspect. Biol..

[163] Kim W., Bennett E.J., Huttlin E.L., Guo A., Li J., Possemato A., Sowa M.E., Rad R., Rush J., Comb M.J. (2011). Systematic and quantitative assessment of the ubiquitin-modified proteome. Mol. Cell.

[164] Bekker-Jensen S., Mailand N. (2010). Assembly and function of DNA double-strand breakrepair foci in mammalian cells. DNA Repair.

[165] Komander D., Rape M. (2012). The ubiquitin code. Annu. Rev. Biochem..

[166] Bergink S., Jentsch S. (2009). Principles of ubiquitin and SUMO modifications in DNA repair. Nature.

[167] Panier S., Durocher D. (2009). Regulatory ubiquitylation in response to DNA double-strand breaks. DNA Repair.

[168] Mailand N., Bekker-Jensen S., Faustrup H., Melander F., Bartek J., Lukas C., Lukas J. (2007). RNF8 ubiquitylates histones at DNA double-strand breaks and promotes assembly of repair proteins. Cell.

[169] Lukas J., Lukas C., Bartek J. (2011). More than just a focus: The chromatin response to DNA damage and its role in genome integrity maintenance. Nat. Cell Biol..

[170] Schwertman P., Bekker-Jensen S., Mailand N. (2016). Regulation of DNA double-strand break repair by ubiquitin and ubiquitin-like modifiers. Nat. Rev. Mol. Cell Biol..

[171] Galanty Y., Belotserkovskaya R., Coates J., Polo S., Miller K.M., Jackson S.P. (2009). Mammalian SUMO E3-ligases PIAS1 and PIAS4 promote responses to DNA double-strand breaks. Nature.

[172] Sun H., Leverson J.D., Hunter T. (2007). Conserved function of RNF4 family proteins in eukaryotes: Targeting a ubiquitin ligase to SUMOylated proteins. EMBO J..

[173] Kusumoto R., Masutani C., Sugasawa K., Iwai S., Araki M., Uchida A., Mizukoshi T., Hanaoka F. (2001). Diversity of the damage recognition step in the global genomic nucleotide excision repair In Vitro. Mutat. Res..

[174] Ulrich H.D. (2009). Regulating post-translational modifications of the eukaryotic replication clamp PCNA. DNA Repair.

[175] Wojcik C., DeMartino G.N. (2003). Intracellular localization of proteasomes. Int. J. Biochem. Cell Biol..

[176] Kouranti I., Peyroche A. (2012). Protein degradation in DNA damage response. Semin. Cell Dev. Biol..

[177] Shi W., Ma Z., Willers H., Akhtar K., Scott S.P., Zhang J., Powell S., Zhang J. (2008). Disassembly of MDC1 foci is controlled by ubiquitin-proteasome-dependent degradation. J. Biol. Chem..

[178] Ohta T., Sato K., Wu W. (2011). The BRCA1 ubiquitin ligase and homologous recombination repair. FEBS Lett..

[179] Ferretti L.P., Himmels S.F., Trenner A., Walker C., von Aesch C., Eggenschwiler A., Murina O., Enchev R.I., Peter M., Freire R. (2016). Cullin3-KLHL15 ubiquitin ligase mediates CtIP protein turnover to fine-tune DNA-end resection. Nat. Commun..

[180] Menendez D., Inga A., Resnick M.A. (2009). The expanding universe of p53 targets. Nat. Rev. Cancer.

[181] Brooks C.L., Gu W. (2011). p53 regulation by ubiquitin. FEBS Lett..

[182] Manfredi J.J. (2010). The Mdm2-p53 relationship evolves: Mdm2 swings both ways as an oncogene and a tumor suppressor. Genes Dev..

[183] Khoo K.H., Verma C.S., Lane D.P. (2014). Drugging the p53 pathway: Understanding the route to clinical efficacy. Nat. Rev. Drug Discov..

[184] Ramadan K., Meerang M. (2011). Degradation-linked ubiquitin signal and proteasome are integral components of DNA double strand break repair: New perspectives for anti-cancer therapy. FEBS Lett..

[185] Hernandez-Pigeon H., Laurent G., Humbert O., Salles B., Lautier D. (2004). Degadration of mismatch repair hMutSα heterodimer by the ubiquitin-proteasome pathway. FEBS Lett..

[186] El-Shemerly M., Janscak P., Hess D., Jiricny J., Ferrari S. (2005). Degradation of human exonuclease 1b upon DNA synthesis inhibition. Cancer Res..

[187] Parsons J.L., Tait P.S., Finch D., Dianova I.I., Edelmann M.J., Khoronenkova S.V., Kessler B.M., Sharma R.A., McKenna W.G., Dianov G.L. (2009). Ubiquitin ligase ARF-BP1/Mule modulates base excision repair. EMBO J..

[188] Parsons J.L., Tait P.S., Finch D., Dianova I.I., Allinson S.L., Dianov G.L. (2008). CHIP-mediated degradation and DNA damage-dependent stabilization regulate base excision repair proteins. Mol. Cell.

[189] Parsons J.L., Dianova I.I., Finch D., Tait P.S., Ström C.E., Helleday T., Dianov G.L. (2010). XRCC1 phosphorylation by CK2 is required for its stability and efficient DNA repair. DNA Repair.

[190] Wang T., Simbulan-Rosenthal C.M., Smulson M.E., Chock P.B., Yang D.C. (2008). Polyubiquitylation of PARP-1 through ubiquitin K48 is modulated by activated DNA, NAD+, and dipeptides. J. Cell Biochem..

[191] Messner S., Schuermann D., Altmeyer M., Kassner I., Schmidt D., Schär P., Müller S., Hottiger M.O. (2009). Sumoylation of poly(ADP-ribose) polymerase 1 inhibits its acetylation and restrains transcriptional coactivator function. FASEB J..

[192] Kurihara Y., Kanki T., Aoki Y., Hirota Y., Saigusa T., Uchiumi T., Kang D. (2012). Mitophagy plays an essential role in reducing mitochondrial production of reactive oxygen species and mutation of mitochondrial DNA by maintaining mitochondrial quantity and quality in yeast. J. Biol. Chem..

[193] Komatsu M., Kurokawa H., Waguri S., Taguchi K., Kobayashi A., Ichimura Y., Sou Y.S., Ueno I., Sakamoto A., Tong K.I. (2010). The selective autophagy substrate p62 activates the stress-responsive transcription factor Nrf2 through inactivation of Keap1. Nat. Cell Biol..

[194] Tanida I. (2011). Autophagy basics. Microbiol. Immunol..

[195] Eliopoulos A.G., Havaki S., Gorgoulis V.G. (2016). DNA damage response and autophagy: A meaningful partnership. Front. Genet..

[196] Mizushima N., Komatsu M. (2011). Autophagy: Renovation of cells and tissues. Cell.

[197] Levine B., Klionsky D.J. (2004). Development by self-digestion: Molecular mechanisms and biological functions of autophagy. Dev. Cell.

[198] Karantza-Wadsworth V., Patel S., Kravchuk O., Chen G., Mathew R., Jin S., White E. (2007). Autophagy mitigates metabolic stress and genome damage in mammary tumorigenesis. Genes Dev..

[199] Song X., Narzt M.S., Nagelreiter I.M., Hohensinner P., Terlecki-Zaniewicz L., Tschachler E. (2017). Autophagy deficient keratinocytes display increased DNA damage, senescence and aberrant lipid composition after oxidative stress In Vitro and In Vivo. Redox Biol..

[200] Jin S. (2006). Autophagy, mitochondrial quality control, and oncogenesis. Autophagy.

[201] Jin S., White E. (2007). Role of autophagy in cancer: Management of metabolic stress. Autophagy.

[202] Lenaz G., Bovina C., D’Aurelio M., Fato R., Formiggini G., Genova M.L., Giuliano G., Pich M.M., Paolucci U.G.O., Castelli G.P. (2002). Role of mitochondria in oxidative stress and aging. Ann. N. Y. Acad. Sci..

[203] Mathew R., Karp C.M., Beaudoin B., Vuong N., Chen G., Chen H.Y., Bray K., Reddy A., Bhanot G., Gelinas C. (2009). Autophagy suppresses tumorigenesis through elimination of p62. Cell.

[204] Rello-Varona S., Lissa D., Shen S., Niso-Santano M., Senovilla L., Mariño G., Vitale I., Jemaá M., Harper F., Pierron G. (2012). Autophagic removal of micronuclei. Cell Cycle.

[205] Ivanov A., Pawlikowski J., Manoharan I., van Tuyn J., Nelson D.M., Rai T.S., Shah P.P., Hewitt G., Korolchuk V.I., Passos J.F. (2013). Lysosome-mediated processing of chromatin in senescence. J. Cell Biol..

[206] Evangelou K., Lougiakis N., Rizou S.V., Kotsinas A., Kletsas D., Muñoz-Espín D., Kastrinakis N.G., Pouli N., Marakos P., Townsend P. (2017). Robust, universal biomarker assay to detect senescent cells in biological specimens. Aging Cell.

[207] Dou Z., Xu C., Donahue G., Shimi T., Pan J.A., Zhu J., Ivanov A., Capell B.C., Drake A.M., Shah P.P. (2015). Autophagy mediates degradation of nuclear lamina. Nature.

[208] Yue Z., Jin S., Yang C., Levine A.J., Heintz N. (2003). Beclin 1, an autophagy gene essential for early embryonic development, is a haploinsufficient tumor suppressor. Proc. Natl. Acad. Sci. USA.

[209] Kuma A., Hatano M., Matsui M., Yamamoto A., Nakaya H., Yoshimori T., Ohsumi Y., Tokuhisa T., Mizushima N. (2004). The role of autophagy during the early neonatal starvation period. Nature.

[210] Mathew R., Kongara S., Beaudoin B., Karp C.M., Bray K., Degenhardt K., Chen G., Jin S., White E. (2007). Autophagy suppresses tumor progression by limiting chromosomal instability. Genes Dev..

[211] Robert T., Vanoli F., Chiolo I., Shubassi G., Bernstein K.A., Rothstein R., Botrugno O.A., Parazzoli D., Oldani A., Minucci S. (2011). HDACs link the DNA damage response, processing of double-strand breaks and autophagy. Nature.

[212] Lin W., Yuan N., Wang Z., Cao Y., Fang Y., Li X., Xu F., Song L., Wang J., Zhang H. (2015). Autophagy confers DNA damage repair pathways to protect the hematopoietic system from nuclear radiation injury. Sci. Rep..

[213] Liu E.Y., Xu N., O’Prey J., Lao L.Y., Joshi S., Long J.S., O’Prey M., Croft D.R., Beaumatin F., Baudot A.D. (2015). Loss of autophagy causes a synthetic lethal deficiency in DNA repair. Proc. Natl. Acad. Sci. USA.

[214] Bae H., Guan J.L. (2011). Suppression of autophagy by FIP200 deletion impairs DNA damage repair and increases cell death upon treatments with anti-cancer agents. Mol. Cancer Res..

[215] Wang Y., Zhang N., Zhang L., Li R., Fu W., Ma K., Li X., Wang L., Wang J., Zhang H. (2016). Autophagy regulates chromatin ubiquitination in DNA damage response through elimination of SQSTM1/p62. Mol. Cell.

[216] Hewitt G., Carroll B., Sarallah R., Correia-Melo C., Ogrodnik M., Nelson G., Otten E.G., Manni D., Antrobus R., Morgan B.A. (2016). SQSTM1/p62 mediates crosstalk between autophagy and the UPS in DNA repair. Autophagy.

[217] Tsakiri E.N., Iliaki K.K., Höhn A., Grimm S., Papassideri I.S., Grune T., Trougakos I.P. (2013). Diet-derived advanced glycation end products or lipofuscin disrupts proteostasis and reduces life span in Drosophila melanogaster. Free Radic. Biol. Med..

[218] Dimopoulos M.A., Moreau P., Palumbo A., Joshua D., Pour L., Hájek R., Facon T., Ludwig H., Oriol A., Goldschmidt H. (2016). Carfilzomib and dexamethasone versus bortezomib and dexamethasone for patients with relapsed or refractory multiple myeloma (ENDEAVOR): A randomised, phase 3, open-label, multicentre study. Lancet Oncol..

